# Gene flow and introgression are pervasive forces shaping the evolution of bacterial species

**DOI:** 10.1186/s13059-022-02809-5

**Published:** 2022-11-10

**Authors:** Awa Diop, Ellis L. Torrance, Caroline M. Stott, Louis-Marie Bobay

**Affiliations:** Department of Biology, University of North Carolina Greensboro, Greensboro, North Carolina, 321 McIver Street, PO Box 26170, Greensboro, NC 27402 USA

**Keywords:** Gene flow, Biological species definition, Introgression, Bacterial evolution, Recombination, Core genome

## Abstract

**Background:**

Although originally thought to evolve clonally, studies have revealed that most bacteria exchange DNA. However, it remains unclear to what extent gene flow shapes the evolution of bacterial genomes and maintains the cohesion of species.

**Results:**

Here, we analyze the patterns of gene flow within and between >2600 bacterial species. Our results show that fewer than 10% of bacterial species are truly clonal, indicating that purely asexual species are rare in nature. We further demonstrate that the taxonomic criterion of ~95% genome sequence identity routinely used to define bacterial species does not accurately represent a level of divergence that imposes an effective barrier to gene flow across bacterial species. Interruption of gene flow can occur at various sequence identities across lineages, generally from 90 to 98% genome identity. This likely explains why a ~95% genome sequence identity threshold has empirically been judged as a good approximation to define bacterial species. Our results support a universal mechanism where the availability of identical genomic DNA segments required to initiate homologous recombination is the primary determinant of gene flow and species boundaries in bacteria. We show that these barriers of gene flow remain porous since many distinct species maintain some level of gene flow, similar to introgression in sexual organisms.

**Conclusions:**

Overall, bacterial evolution and speciation are likely shaped by similar forces driving the evolution of sexual organisms. Our findings support a model where the interruption of gene flow—although not necessarily the initial cause of speciation—leads to the establishment of permanent and irreversible species borders.

**Supplementary Information:**

The online version contains supplementary material available at 10.1186/s13059-022-02809-5.

## Background

Gene flow is a key evolutionary process upon which theoretical frameworks of speciation have been primarily founded in sexual organisms. In contrast, bacteria reproduce asexually, and developing a theoretical framework to establish a definition of bacterial species has proven difficult; and some have even questioned the existence of prokaryotic species [1, 2]. As a result, most studies rely on operational definitions of bacterial species, which are often based on arbitrary sequence thresholds [3, 4]. Albeit convenient, these definitions impede our ability to fully understand the evolution and dynamics of bacteria. Some bacteria can engage in gene flow via homologous recombination [5] and this observation has led a growing number of researchers to suggest that bacterial species and speciation might be best defined using the same evolutionary theory developed for sexual organisms [6–11]; the biological species concept (BSC) [12–14]. It has been long established that even distantly related bacteria can occasionally exchange genes through horizontal gene transfers (HGT), but this process usually involves accessory genes that are not part of the genomic backbone—the core genome—of the species. The core genome, representing the most functionally important set of genes, is thought to evolve primarily vertically [15] and thus, is the focus of most efforts to understand the population structure and evolution of bacteria. Despite many years of work, the number of bacterial species engaging in gene flow and the limits of gene flow between populations and species remain poorly defined. Therefore, although bacteria are often presumed to evolve clonally, the prevalence of truly clonal species remains unknown [5]. Additionally, some bacterial species appear "fuzzy" as ongoing gene flow can be maintained between the core genomes of rather distant species and these processes might be analogous to the patterns of introgression frequently observed in sexual organisms [16–18]. Understanding the impact of DNA flux and developing a theory-anchored bacterial species concept remains a fundamental gap in evolutionary biology and microbiology. Key to this problem is our ability to recognize (i) which populations do and do not engage in gene flow, (ii) which bacterial species are truly clonal, and (iii) to what extent distant species can engage in gene flow.

Here we analyzed the patterns of gene flow *within* and *between* species across >2600 bacterial species and >30,000 genomes. We identified which of these bacterial species are truly clonal, classified bacteria into biological species by redefining species boundaries based on gene flow, and analyzed the patterns of gene flow between species (i.e., introgression). We show that very few bacterial species (2.6%) are unambiguously clonal, suggesting that truly asexual lineages are extremely rare across the Tree of Life. Our results also indicate that introgression is frequent in bacteria and that genomic divergence is the main factor determining the frequency of introgression events between species. Overall, our findings support a universal model of sexual isolation in bacteria where the decreasing frequency of identical DNA segments—which are required to initiate homologous recombination—appears to be the primary determinant of the interruption of gene flow. It further suggests that this mechanism could lead to the establishment of permanent species barriers between bacterial populations.

## Results and Discussion

### Very few bacteria are truly clonal

**Fig. 1:**
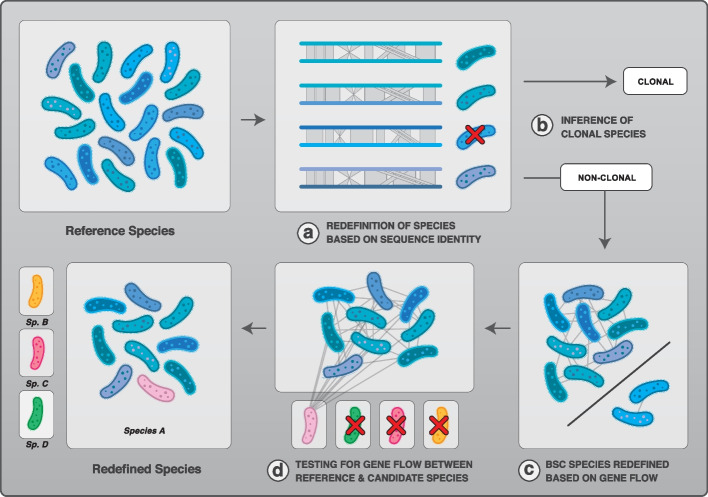
Approach used to reclassify bacterial species. The set of complete genomes for each species containing ≥15 genomes was analyzed and used as reference species. **a** Each reference species was redefined based on sequence identity thresholds using the ANI of core genes metric, i.e., the pairwise identity score computed on the core genome of each species. Genomes were considered as part of the same species when sharing at least 94% ANI of core genes. **b** Clonal species were inferred based on simulations without gene flow and based on Linkage Disequilibrium analysis. Reference species inferred as clonal by at least one of these methods were excluded from the next steps of the analysis. **c** Reference species were redefined into BSC species based on gene flow: genomes that showed a significant reduction in gene flow with the rest of the population were excluded from the reference species. **d** The redefined reference species based on steps B and C were tested for gene flow against candidate species. Candidate species were selected as species related to the reference species based on either taxonomic nomenclature (i.e., same genus name) or based on sequence relatedness. In this theoretical example, four candidate species were tested for gene flow against the reference species. One candidate species was found to engage in gene flow with the reference species and those were reclassified as the same species (species A). The three other candidate species were not found to engage in gene flow with the reference species and their original classification was kept (species B, C, and D)

Bacteria reproduce asexually but are known to frequently engage in homologous recombination. Despite many studies on recombination, it remains unclear *how many* and *which* bacterial species can be considered truly clonal in nature. Here we addressed this question by analyzing signals of recombination across a large set of bacterial species. We first reclassified all bacterial species for which at least 15 complete genomes have been sequenced using sequence identity thresholds commonly used to define bacterial species. To do this, we used the Average Nucleotide Identity (ANI) among the core genes with a cutoff value of 94% core-genome identity to reclassify named bacterial species into ANI-redefined species (Fig. 1a, Additional file 1: Table S1) [19]. The species containing less than 15 genomes after redefinition were excluded and this step yielded a total of 227 ANI-redefined species. To identify clonal species (Fig. 1b), we first analyzed the patterns of homoplasic alleles (*h*) relative to non-homoplasic alleles (*m*). Homoplasic alleles are those whose distribution is incompatible with a scenario of vertical inheritance from a single common ancestor. They are likely the result of recombination events, but some can also accumulate due to independent convergent mutations. Thus, clonal species are expected to present low *h/m* ratios, but the exact number of homoplasic alleles found depends on the rate of substitutions, the age since divergence, and substitution biases. Therefore, we simulated the evolution of each species (*n*=227) under a model of purely clonal evolution with substitution parameters that closely mimic the dataset for each species. We then estimated the *h/m* ratio for each species and the corresponding set of simulated genomes. Truly clonal species are expected to display *h/m* ratios similar to the simulated genomes, whereas recombining species should show higher *h/m* ratios when compared to the simulations (Additional file 2: Fig. S1a). Next, we used the patterns of Linkage disequilibrium (LD) to infer clonality. In recombining genomes, LD (measured by *r*^*2*^) decreases relative to genomic distances between the two loci (Additional file 2: Figs. S1b, d). Due to the absence of recombination, clonal species should not exhibit a significant decrease between *r*^*2*^ and genomic distances (Additional file 2: Fig. S1b-c).

Overall, we inferred that only 9.7% and 5.7% of species were clonal based on *h/m* ratios and LD respectively, with 2.6% of clonal species being inferred by both methods and a total of 12.8% inferred by at least one method (Additional file 2: Fig. S2). Importantly, we noted that the species that we predicted as clonal did not present significantly lower amounts of polymorphisms (Additional file 2: Fig. S2c) relative to the non-clonal ones, suggesting that lower statistical power and the accuracy of parameter estimation did not substantially bias our analysis. Our results indicate that most bacterial species display clear signs of recombination and as little as 2.6% appear truly clonal (Additional file 3: Table S2). Species inferred as purely clonal were often endosymbionts from the genera *Chlamydia*, *Brucella,* and *Bordetella*, which have previously been defined as clonal [20–22]. However, not all endosymbiont species were found to be systematically clonal (Additional file 3: Table S2).

Previous works have noted that adaptive evolution can sometimes lead to the presence of convergent mutations [23–25]. Although these observations were usually made in genes facing very strong selective pressures (e.g., antibiotic resistance genes) and/or during experimental evolution studies [23] we tested whether adaptive evolution could be responsible for the accumulation of a substantial fraction of the homoplasies detected in bacterial core genomes. We reasoned that if adaptive evolution were responsible for the presence of many homoplasies, those would be predominantly observed at non-synonymous sites where opportunities for positive selection are much higher than at synonymous sites. Indeed, synonymous sites are evolving under weak selective pressures which are primarily attributed to codon usage and nucleotide composition [26] and are typically acting at the scale of the entire gene and not at a particular site. For each species, we estimated the fractions of homoplasic and non-homoplasic alleles that were found at synonymous and non-synonymous positions across core genomes. Our results indicate that >50% homoplasic alleles are found at synonymous sites (Additional file 2: Fig. S3), and, importantly, we observed that homoplasic alleles were not found more frequently at non-synonymous sites when compared to non-homoplasic alleles. In fact, the opposite trend was observed: homoplasic alleles are more frequently synonymous when compared to non-homoplasic alleles for ~95% of species (Additional file 2: Fig. S3). Therefore, since the distribution of homoplasic alleles is not biased toward non-synonymous positions, adaptive selection is unlikely to explain the presence of homoplasies in the core genome of these species. Although this analysis does not completely exclude the possibility that some homoplasies are the result of positive selection, it should be noted that the homoplasies that we inferred are pervasive across bacterial core genomes with an average of 65,670 homoplasies per reference species (Additional file 3: Table S2). This represents an average of 35 homoplasies per core gene across reference species (Additional file 3: Table S2), indicating that our signal of gene flow is not driven by a few isolated genes. This strongly supports the view that positive selection plays a negligible role in the observed patterns of homoplasies.

Our results show that bacteria—like eukaryotes—present very few truly asexual lineages. As hypothesized for eukaryotes [27] truly asexual species of bacteria could be short-lived and therefore rare in nature (e.g., due to Muller’s ratchet). In contrast, *Buchnera aphidicola* and other insect endosymbionts are well-studied cases of strictly clonal bacteria that have co-evolved within their insect hosts for millions of years [28] but those were not included in this study due to the scarcity of genomes available and the parameters used to define initial species boundaries. It is likely that these ancient clonal lineages managed to escape extinction—i.e., to prolong their existence—by making themselves indispensable for their hosts by synthetizing several essential amino acids absent from the diet of sap-feeding insects, although these bacteria can eventually be replaced by new symbionts [29]. In summary, clonal bacteria are likely short-lived in nature, but some symbiotic species might be able to avoid extinction for substantially longer periods of time by evolving into organelle-like entities.

### Gene flow delineates biological species boundaries

**Fig. 2:**
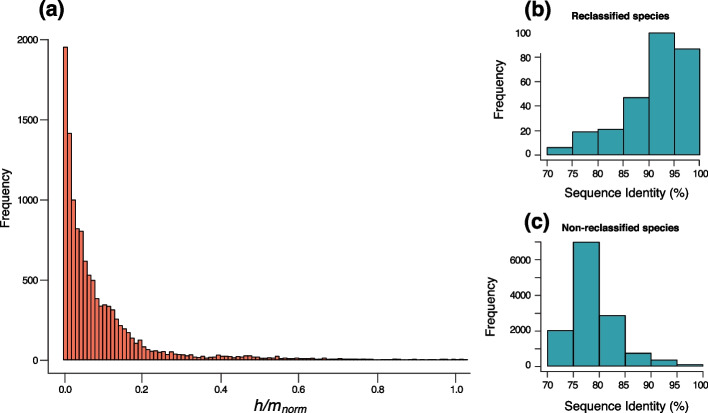
Patterns of gene flow across species. **a** Distribution of adjusted *h/m* ratios between candidate and reference species. The adjusted ratio of homoplasic to non-homoplasic alleles *h/m*_*norm*_ was computed to quantify gene flow. *h/m*_*norm*_ was computed by adjusting the values of *h/m* on the candidate and reference species (*h/m*_*cand*_) relative to *h/m* of the reference species alone (*h/m*_*ref*_) and relative to the *h/m* ratio expected to result from convergent mutations alone (*h/m*_*0*_). The adjusted ratio *h/m*_*norm*_ is expected to be null when *h/m*_*cand*_ is equal to *h/m*_*0*_ (*h/m*_*cand*_ = *h/m*_*0*_). The adjusted ratio *h/m*_*norm*_ is expected to be near unity when *h/m*_*cand*_ is similar to *h/m*_*ref*_ (*h/m*_*cand*_ = *h/m*_*ref*_). Only values ranging between 0 and 1 were represented (several datapoints exceeded 1, *n*=191, 1.6%). **b** Distribution of maximum sequence identities (ANI of core genes) between candidate and reference species that were reclassified as part of the same species based on gene flow. **c** Distribution of maximum sequence identities (ANI of core genes) between candidate and reference species that were not reclassified as part of the same species based on gene flow

We reclassified bacteria based on gene flow by comparing patterns of recombination *within* and *between* species. We have previously developed an approach that was shown to identify the presence and the interruption of gene flow in bacteria as well as in sexual organisms [9, 30]. First, we reclassified our set of non-clonal species (*n*=198) based on gene flow (Fig. 1c), as in [9, 30], by excluding genomes showing a significant drop in *h/m* ratio (*within* species redefinition). We redefined ~25% of species, which contained some non-recombining genomes and those were excluded from the species, yielding a total of 197 biological species (BSC species) (Additional file 1: Table S1). The redefined species were then used as the set of "reference species" against which other species were compared (*between* species comparison). Briefly, we selected every candidate species related to at least one of the reference species of our dataset and analyzed patterns of gene flow against each of the related reference species (Fig. 1d). The candidate species were selected by downloading all named species within the same genus of the reference species available on NCBI, and also closely related species from other genera (see the Methods section). Following this process, we analyzed a total of 2446 candidate species by building the set of core genes shared between each candidate species and each reference species. Each pair of reference/candidate species was analyzed for gene flow by computing *h/m* ratios and conducting a resampling analysis to test the robustness of our metric. Although gene flow varies to some extent along a species’ core genome, these fluctuations are rather modest [31, 32] and our sets of core genomes were typically composed of hundreds to thousands of genes for each pair of reference/candidate species (Additional file 4: Table S3), thereby ensuring robust genome-wide estimates. A fraction of homoplasies can be introduced by convergent mutations and those are expected to increase in frequency as substitutions accumulate during genome divergence. Therefore, for all pairs of reference/candidate species, simulated genomes were evolved *in silico* without recombination and with similar characteristics and divergence rates as the candidate species relative to the reference species. Each simulated candidate species was then used to compute the ratio *h/m*_*0*_ which represents the *h/m* ratio expected to result from convergent mutations alone since the divergence from the last common ancestor shared with the reference species. Using this procedure, we computed the adjusted ratio *h/m*_*norm*_ for each pair of reference/candidate species. This metric corresponds to the *h/m* ratio adjusted for the amount of homoplasies expected to result from convergent mutations (*h/m*_*0*_) and the amount of homoplasies estimated in the reference species alone (*h/m*_*ref*_). If the candidate and the reference species are freely engaging in gene flow, the *h/m* ratio should be very similar to *h/m*_*ref*_ (*h/m*_*norm*_ = 1). In contrast, if the two species do not engage in gene flow at all, the *h/m* ratio should be very similar to *h/m*_*0*_ (*h/m*_*norm*_ = 0). As expected, we observed that most candidate species do not engage in gene flow with the corresponding reference species (Fig. 2a, Additional file 2: Fig. S4 and Additional file 4: Table S3) as most *h/m*_*norm*_ values were close to 0. However, we identified that 11.3% of all candidate species unambiguously engage in gene flow with other species (*h/m* is not significantly lower than *h/m*_*ref*_ and is significantly higher than *h/m*_*0*_), indicating that these species pairs can be considered a single BSC species. Among reclassified species appeared well-documented cases of ambivalent taxonomy such as *Escherichia coli* and *Shigella*, which have been suggested to be classified into the same species based on sequence identity thresholds and phylogenetic analyses [33–35]. Moreover, we inferred that the species of the *Burkholderia cepacia* complex (Bcc)—a group of ubiquitous opportunistic pathogens which has undergone frequent taxonomic changes [36, 37]—constitute a single biological species. The species of the Bcc complex are all closely related, with at least 90% ANI of core genes. Overall, the vast majority of all redefined BSC species in our dataset were closely related, i.e., typically >90% ANI of core genes (Fig. 2b, c, Additional file 4: Table S3). A few redefined species were distantly related (<80%) but displayed weak signals of gene flow (Additional file 2: Fig. S5).

### Pervasive introgression across bacteria

**Fig. 3:**
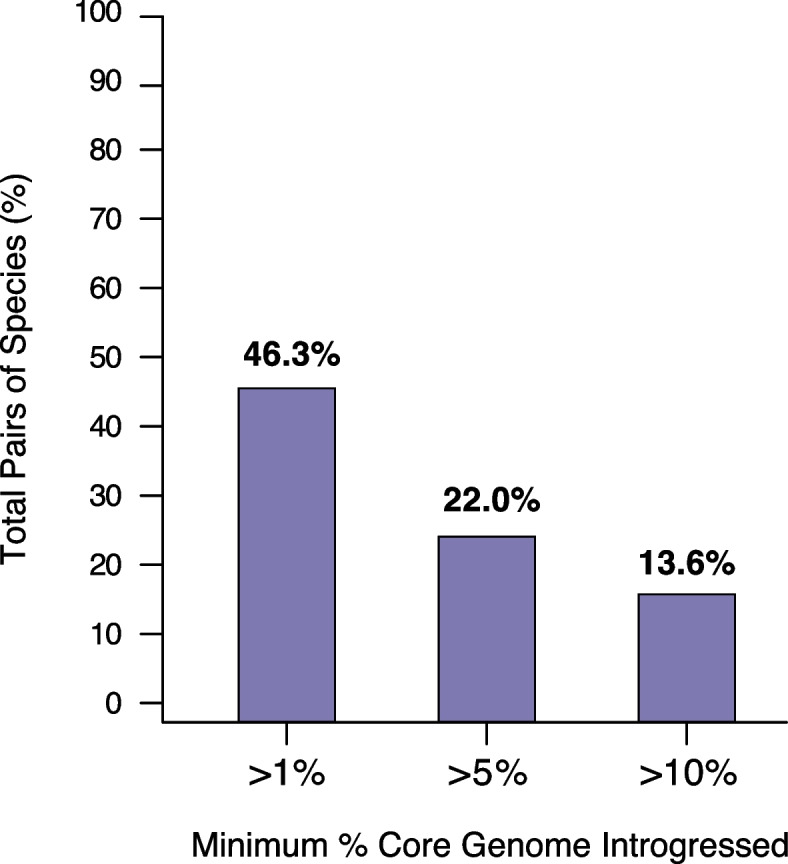
Levels of introgression across species. The graph represents the inferred fraction of candidate/reference species pairs presenting over 1%, 5%, and 10% of introgressed DNA in their core genome, respectively

We reclassified several species into a single BSC species based on the presence/absence of gene flow. However, the analysis of homoplasies revealed that many species pairs present intermediate levels of *h/m* ratios (Fig. 2a) which are neither compatible with similar levels of gene flow observed in the reference species nor with the complete absence of gene flow. This raises the following question: Although two groups of genomes may be classified into distinct species due to lowered exchanges of gene flow, to what extent can they maintain some level of gene flow? Measuring gene flow based on *h/m* ratios cannot unequivocally address this question. Indeed, our *h/m* metric can retain some of the signal of gene flow even though gene flow might no longer be ongoing between two sets of genomes. Namely, intermediate levels of gene flow, as measured by *h/m* ratios, might not represent reduced ongoing gene flow between two species, but rather could reflect a complete—but recent—interruption of gene flow. To address this question, we derived a metric to quantify gene flow between candidate and reference species that were not reclassified as part of the same species and we named this metric *introgression score (S*_*i*_*)*. For each of the 13,437 pairs of candidate/reference species (Additional file 4: Table S3), we ran a 100bp scanning window along the core genome concatenate. Each 100bp fragment was defined as a transfer between the two species if at least one genome of the reference species was more similar to the candidate species than another genome of the reference species. The introgression score was then defined as the percent of the core genome that has been exchanged between the candidate and the reference species. Quantifying gene flow with this approach revealed a clear positive correlation between *S*_*i*_ and *h/m* (Additional file 2: Fig. S6), but many candidate/reference species pairs exhibited a lower introgression score relative to *h/m*, as predicted based on the fact that *h/m* can retain older signals of gene flow. Most candidate/reference species pairs were inferred to present rather modest amounts of introgressed DNA in their core genome (Fig. 3). The amount of gene flow inferred by *S*_*i*_ varied widely across candidate/reference species pairs, but on average, 5.2% of the core genome was found to be exchanged between pairs of species with 46.3% of all species pairs presenting >1% of introgressed DNA (Fig. 3, Additional file 4: Table S3).

Interestingly, we observed uneven levels of introgression across lineages. Species of the classes *Alpha-Proteobacteria* and *Beta-Proteobacteria* displayed some of the highest levels of introgression, whereas species of the *Spirochaetia*, *Clostridia*, and *Chlamydiia* showed very little signs of introgression (Additional file 2: Fig. S7). Several species in our dataset were previously found to have exchanged DNA through introgression: *Campylobacter jejuni* and *C. coli*, which only share 85% sequence identity, were shown to display up to 23% of introgression in their core genome [18] for a single pair of strains. Our estimates revealed that 29% of the core genome of *C. jejuni* contains introgressed sequences from *C. coli* (cumulatively across all strains), which is in close agreement with the previous estimate. The *Neisseria* genus represents another interesting case of porous species boundaries. The species of this genus were previously reported to engage extensively in genetic exchange and this had been attributed to the presence of numerous *Neisseria* species living in the same ecological niches in the human body [16, 38–40]. In agreement with previous studies, our results show that *N. meningitidis* and *N. lactamica—*which both colonize the human nasopharynx—share higher levels of introgressed DNA in their core genes relative to non-pathogenic *Neisseria* species. In contrast, we inferred that 32% of the core genome of *N. meningitidis* contains introgressed sequences from its closest relative *N. gonorrhoeae* although these two species do not typically cohabit the same ecological niche [40, 41]. These findings indicate that many species appear to engage in introgression in a manner that is not always in agreement with their known ecology, providing new insights into bacterial evolution and speciation [18, 42, 43]. Overall, our results indicate that introgression is a pervasive process shaping the evolution of the core genome of bacterial species.

**Fig. 4:**
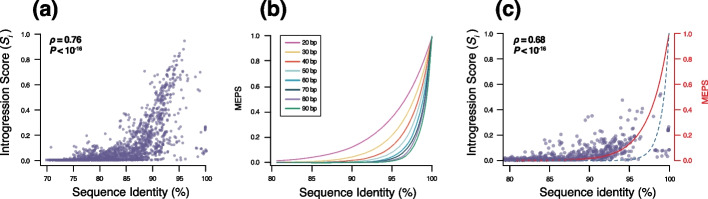
Introgression between species and MEPS. **a** Introgression scores (*S*_*i*_) between candidate and reference species that were not reclassified as part of the same species. *S*_*i*_ represents the fraction of the core genome that shows evidence of introgression between the candidate species and at least one genome of the reference species. Introgressed regions were defined as 100bp fragments that were more similar to the candidate species than at least one of the genomes of the reference species. Only introgressed fragments with ≥95% sequence identity between the candidate and reference species were considered as introgressed. Coefficient correlation *ρ* and *P*-value were estimated with Spearman’s rank correlation test. **b** Simulated frequency of identical Minimal Efficient Processing Segments (MEPS) expected relative to sequence identity. Different lines represent MEPS ranging from 20 to 90bp. **c** Introgression scores (*S*_*i*_) computed for recent introgression events. *S*_*i*_ was computed as described above between candidate and reference species that were not reclassified as part of the same species. Only introgressed fragments presenting 100% sequence identity between the candidate and reference species were considered as introgressed. The red line corresponds to a MEPS size of 40bp and the dashed blue line represents the expected number of 100bp identical fragments for a pair of genomes

The amount of transferred DNA between species’ core genomes was found to vary extensively based on sequence divergence (Fig. 4a). A minority of species’ pairs showed little to no signs of introgression despite sharing high sequence identity, e.g., >90% (Fig. 4a). In contrast, most species display a positive exponential relationship between sequence relatedness and levels of introgression (Fig. 4a). The same trend was observed when using different identity thresholds to define introgressed fragments (Additional file 2: Fig. S8). Note that our method was designed to avoid inferring identical fragments as introgression when they actually result from vertical evolution, and only strains that are more closely related to one another rather than to the candidate species were considered for this analysis. The amount of vertically inherited fragments falsely inferred as introgression is expected to be negligible, but the exact number depends on the levels of divergence between species and on the levels of polymorphisms of each species. For simplicity, the number of identical 100bp segments found between simulated genome pairs was generated to represent the maximal theoretical number of DNA segments that could result from vertical evolution (Fig. 4c, dashed blue line). We observed that most species pairs present higher levels of identical DNA segments than expected based on our upper limit of vertically inherited segments (Fig. 4c). Findings indicate that, despite a clear reduction in gene flow, many species still engage in some levels of DNA exchange in their core genomes and that the frequency of introgression is intimately related to sequence divergence.

### A model of gene flow shaping species boundaries

Several studies have experimentally quantified recombination rate relative to sequence divergence in *Escherichia*, *Bacillus* and *Streptococcus* and a similar exponential relationship was observed [44–47]. One proposed hypothesis [48] was based on the observation that homologous recombination requires the presence of nearly identical DNA fragments called MEPS (minimal efficient processing segment) to initiate recombination [49]. The size of the MEPS has been experimentally shown to vary between 25 and 90bp across species [49]. We simulated multiple sets of genomes with different levels of divergence and identified the frequency of different-sized MEPS (Fig. 4b). We found that the observed relationship between introgression and sequence divergence could be explained by the availability of MEPS. A MEPS size of 40bp likely represents the average size required to initiate recombination across species (Fig. 4c, red line), although MEPS size likely varies across lineages. A simple model might account for the overall patterns of introgression observed across bacteria: as genomes diverge, the density of MEPS available to initiate recombination decreases. Due to the exponential shape of this relationship, gene flow appears to be sharply reduced when genomes reach 2–10% of sequence divergence. This level of genome divergence approximately corresponds to the threshold of ~95% genome sequence identity that is commonly used to define bacterial species [4, 50, 51]. This finding may explain why 95% ANI is commonly judged an adequate threshold to define species boundaries as a sequence identity of roughly 95% effectively interrupts gene flow and could therefore disrupt the genetic cohesiveness in most species. However, the exact sequence identity that confers an effective interruption of gene flow appears to vary across species (Fig. 2b, c), typically ranging from 90 to 98% sequence identity and this is likely due to various MEPS sizes required to initiate homologous recombination across species. These results further support some key aspects of the fragmented speciation model [52], where different regions of the genomes may undergo independent genetic isolation due to sequence divergence and ecological adaptation, which would eventually lead to the interruption of gene flow.

## Conclusions

Biological bacterial species can be defined based on the signal of gene flow, and we argue that this constitutes a theoretical framework upon which a biologically-relevant species concept of bacteria can be built. Defining bacterial species is key to many studies such as those focusing on population genetics, ecology, and pan-genome evolution. Although some bacterial species appear to be truly asexual, those are likely as rare and as short-lived as in multicellular eukaryotes [27]. A ~95% genome sequence identity threshold is commonly used as an approximation to define bacterial species and our results indicate that this threshold appears to vary from 90 to 98% sequence identity across lineages when defining species based on gene flow. Our results may therefore provide an explanation for why the ~95% genome sequence identity threshold has been judged a good approximation in many studies to define species based on empirical observation [4, 50, 51]. Previous studies reported an exponential relationship between homologous recombination and sequence divergence in several bacterial species using experimental settings [44–47]. Our results indicate that the availability of MEPS is likely a universal feature shaping gene flow and speciation in bacteria. Many works have emphasized the role of ecology and selection in bacterial speciation [42, 43] and our findings do not challenge these results. Although niche specialization, physical barriers, and selection might be the primary causes of speciation in bacteria, our study suggests that barriers of gene flow might only become effective and irreversible once genomes have reached a certain level of divergence.

## Methods

### Datasets

All analyzed genomes were downloaded from the GenBank database ftp.ncbi.nlm.nih.gov/genomes/ (September 2018). All named species—as named on GenBank—with ≥15 completely assembled genomes were downloaded. This dataset initially included 84,078 bacterial and archaeal genomes from 331 named species according to species designations on the NCBI website (Additional file 1: Table S1). Protein-coding genes of each genome were extracted based on the annotations. From the original set of 331 named species, only those represented by at least 15 genomes remaining after filtering for missing or incomplete annotations were conserved (Additional file 1: Table S1). Six species presented very large genomic data (>2,500 genomes), i.e., *Acinetobacter baumannii*, *Escherichia coli*, *Klebsiella pneumoniae*, *Salmonella enterica*, *Staphylococcus aureus*, and *Streptococcus pneumoniae*, and 500 genomes were therefore randomly selected for each of these named species in order to reduce the computational load (Additional file 1: Table S1). This total database resulted in a total of 30,694 genomes from 247 named species with ≥15 genomes for each species represents our dataset of *reference species* (Additional file 5: Dataset S1), against which related species—*candidate species*—have been compared. Note that our overall dataset contained a single archaeal species and we therefore referred to our dataset as “bacteria” instead of “prokaryotes” to avoid generalization to all prokaryotes since our dataset includes a single archaeal species. We then downloaded one fully assembled genome sequence and the corresponding annotations for 2595 candidate species (Additional file 6: Dataset S2). Candidate species were selected by choosing species within the same genus of the reference species (e.g., since *Bacillus cereus* was present in our list of reference species, all other *Bacillus* species were used as candidate species). Finally, because bacterial classification can be inconsistent, we also identified genera sharing high sequence identity with one another using a set of 44 universally conserved proteins as in [53] and pairwise distances were computed using RAxML v8 with the PROTGAMMAAUTO option [54]. Genera sharing ≤5% protein sequence distances were considered as potentially misclassified genera. For each reference species of these genera, all the species from the related genera were also used as candidate species. Two groups of genera were found highly related: (i) *Citrobacter*, *Enterobacter*, *Escherichia*, *Klebsiella*, *Salmonella*, and *Shigella* and (ii) *Mycobacterium* and *Mycobacteroides*.

### Definition of core genomes of the reference species

For each reference species, the core genome was built using *CoreCruncher* as previously described [55] with *Usearch* Global v8.0 [56] and the stringent option. *CoreCruncher* was used because it is fast and because it includes a test to exclude potential paralogs and xenologs from the core genome. Orthologs were defined with >70% protein sequence identity and >80% sequence length conservation and all other parameters were set to default. The core genome was defined as the set of single-copy orthologs found in at least 85% of the genomes within each species. Protein sequences of each core gene were then aligned using *Muscle* v3.8.31 [57] with default parameters. Because *Muscle* was unable to align large sequence files, *Mafft* v7.407 [58] was used for the species containing ≥1000 genomes. Protein alignments were then reverse-translated into their corresponding nucleotide sequences. Finally, the nucleotide alignments of all the core genes of each named species were concatenated into a single large alignment as previously described [59].

### Definition of species based on ANI of core genes and gene flow

The core genome concatenates of each of the 247 reference species were used to estimate the ANI of core genes for all genome pairs. This method differs from more traditional ANI, e.g., using FastANI [4], in the fact that traditional ANI represents the average nucleotide identity of all orthologous genes shared by any two genomes while the ANI of core genes represents the average nucleotide identity of the core genes shared by a pair of genomes. The methods are very similar in concept, but the ANI of core genes is a slightly more stringent metric as core genes are usually evolving slower than accessory genes [19]. Pairwise ANI of core gene values were computed using the *distmat* tool of EMBOSS version 6.6.0.0 [60], which calculates the pairwise nucleotide identities from the alignment as previously described [19]. Then, single linkage clustering was performed as described in [19]: all genome pairs with an ANI of core gene similarity cutoff of 94% or higher were joined together and clustered into de novo species (ANI species) (Additional file 1: Table S1).

Gene flow was then inferred using the distance-based method implemented in *ConSpeciFix* (8). Briefly, the matrix of pairwise distances *D* was built using RAxML v8 [54] with the GTR + GAMMA model for each core genome concatenate. This matrix of distances was used to infer homoplasic alleles (*h*) and non-homoplasic alleles (*m*) as described in (8). A genome resampling analysis was conducted for each dataset in order to identify the presence of potential outliers, i.e., genomes that do not engage in gene flow with the rest of the population. Groups of genomes were considered part of the same biological species when found to engage in gene flow, whereas genomes whose inclusion led to a substantial and significant drop in gene flow (*h/m*) based on the exclusion criterion were excluded from the biological species as previously described [9, 30]. First, gene flow was estimated using the dataset of reference species. From each of these named species, genomes that led to a substantial drop in gene flow were identified by a significant and substantial decline in *h/m* ratios (Wilcoxon test, *P*< 0.0001). These genomes were then removed from the dataset, and all remaining genomes were considered members of the same BSC-defined species.

Our final classification of reference species was based on the results of both methods—ANI of core genes and gene flow delimitation. Genomes that were excluded by at least one of these methods were excluded from the reference species. The final dataset of redefined reference species was composed of more than 30,000 genomes across 227 species (Additional file 5: Dataset S1, Additional file 1: Table S1). This dataset is referred as redefined BSC species (but the ANI of core genes was also used to redefine species boundaries). In addition, when named species regrouped more than one cluster with ≥15 genomes, the cluster containing the reference genome on NCBI was selected to represent the reference species and one genome of the rest of the other clusters in the same named species was added to the dataset of candidate species in case it would be reclassified as a part of a different reference species.

### Inference of clonal species

#### Simulation approach

For each species, simulations were conducted in the absence of recombination to estimate the expected number of homoplasies introduced by convergent mutations. The goal of this analysis was to determine which species present *h/m* ratios compatible with a purely clonal model of evolution. First, a maximum likelihood phylogenetic tree for each of the 227 species was built using the total core genome alignment of each species using RAxML v8 [54] with a GTR + Gamma model. Several summary statistics were extracted from the alignment and from the tree for each species: GC-content, core genome alignment length, average pairwise nucleotide diversity (*π*), levels of polymorphisms across codon positions and the transition/transversion ratio (*κ*). We then used *CoreSimul* [61] to generate forward-in-time simulations of the core genome of each species using the parameters specific to each species. Simulations were initiated by generating a random core genome using the length and the GC content estimated for each species. The topology and the branch lengths of the tree were used to simulate core genome evolution without recombination (recombination rate *ρ* was set to zero) under a K2P and codon model using species-specific parameters: the *κ* parameter estimated for each species and the relative levels of polymorphisms estimated across the three codon positions were used to simulate substitutions with specific rates across the three codon positions specific to each species. Phylogenetic programs infer homoplasic alleles as independent mutations, although they often result from recombination events [61]. Therefore, the branch lengths of the trees systematically overestimate the amount of polymorphisms present in the genomes when recombination is present [61, 62]. To address this issue, the genome simulations of each species were conducted multiple times (*n*=99) with different rescaling coefficients as explained in [62]. We then estimated the pairwise ANI of core genes for each simulation replicate and these values were compared to the pairwise ANI of the real core genome of the corresponding species. For each species, the simulated replicate presenting the most similar nucleotide diversity to the real dataset was then selected as the most realistic simulation. Finally, this simulated set of genomes was used to compute the *h/m* ratio for each species. This *h/m* ratio then was used to infer the amount of homoplasies expected under clonal evolution specific to each species. The real *h/m* ratios were compared to the *h/m* ratios of the datasets simulated without recombination to infer which species were clonal (Additional file 2: Fig. S1a, Additional file 3: Table S2). Assuming that all the species in our dataset were strictly clonal, we would expect the real and simulated ratios to be very similar (*y=x*). Most species presented clearly different *h/m* ratios between real and simulated data (Additional file 2: Fig. S1a, Additional file 3: Table S2). Using a resampling analysis, the real *h/m* ratio was re-computed 100 times for each species by excluding exactly one genome for each resampling. The standard deviation of the *h/m* ratios (SD) calculated with the resampling analysis—based on the standard deviation—was used to define the limit between clonal species and non-clonal species (Additional file 2: Fig. S1a, Additional file 3: Table S2). Very similar numbers of species were defined as clonal when using a threshold of 2.SD, 3.SD or 4.SD. The more conservative threshold of 3.SD was then selected to define clonal species with this approach.

#### Linkage disequilibrium approach

Linkage disequilibrium (LD) analysis was conducted in the core concatenate of each redefined reference species as for the simulation study. We used the presence or absence of LD signal to infer the clonality for each of the 227 species. LD was measured using *r*^*2*^ between all pairs of biallelic loci A/a and B/b as: *r*^*2*^ = $\frac{{\left( pAB- pA. pB\right)}^2}{pA.\left(1- pA\right). pB.\left(1- pB\right)}$ with *p*_*AB*_ the proportion of haplotypes AB, *p*_*A*_ the proportion of alleles A, *p*_*B*_ the proportion of alleles B and such as *p*_*a*_*=1-p*_*A*_ and *p*_*b*_*=1-p*_*B*_. Positions in the alignments with ≥25% missing sites due to indels were not included. Because singletons can lead to substantially underestimate the signal of recombination, biallelic sites were only included in the analysis when the least frequent allele was found in at least two genomes. LD was estimated in the core genome concatenate of each redefined reference species using a scanning window of 1,000bp. We considered that a significant decrease in *r*^*2*^ relative to genomic distance between alleles was indicative of the presence of gene flow, whereas the absence of correlation between *r*^*2*^ and genomic distances was indicative of the absence of gene flow. Correlations were assessed with Spearman’s *ρ* and a conservative *p*-value threshold of *P*<0.0001—which accounts for multiple testing (*α*=0.0226)—was defined to infer significant correlations (Additional file 2: Fig. S1b).

### Inference of synonymous and non-synonymous alleles

To test for the potential impact of adaptive evolution on the prevalence of homoplasies, we classified homoplasic alleles (*h*) and non-homoplasic alleles (*m*) as synonymous or non-synonymous across the core genome of all reference species. The core genome of each reference species is a concatenate of genes that were reverse transcribed from protein alignments (see above) and our inference of homoplasies further provide the position of these alleles along the concatenate. To avoid ambiguities, we focused our analysis on codons with a single polymorphic site. The analysis was further restricted to codons whose polymorphic site was bi-allelic, also to avoid ambiguous inferences. We then computed the number of homoplasic alleles and non-homoplasic alleles found at synonymous and non-synonymous positions, respectively. Because reference species vary in core genome size and levels of polymorphisms, we reported the results for the species where ≥100 homoplasic alleles and ≥100 non-homoplasic alleles could be analyzed.

### Definition of the core genome of the reference and candidate species

We used the *ConSpeciFix* pipeline [30] to build the core genome shared between each reference species and each related candidate species (i.e., the species belonging to the same genus or to a closely related genus, see above) using a single randomly selected genome for the candidate species. Briefly, *ConSpeciFix* compares each core gene of the reference species to the genome of the candidate species using *Usearch* Global v8.0 [56] with ≥70% protein identity and ≥80% sequence length conservation. Orthologs were defined as best bidirectional hits and considered part of the shared core genome if found as single-copy as in [9]. Protein sequences of each core genes were aligned and reverse-translated into nucleotides as described above. The shared core genome of each pair of reference + candidate species was then concatenated into a single large alignment. This step resulted in a total of 13,209 core genome concatenates (Additional file 4: Table S3), each corresponding to a specific pair of reference + candidate species. We also calculated the ANI of core genes for all pairs of genomes between reference and candidate species using the same approach as described above.

### Detection of gene flow between species

We tested for the presence of gene flow between each of the BSC-defined species and each candidate species. For each comparison of a candidate species against a BSC-defined reference species, the core genome concatenate for the reference + candidate species was used to infer gene flow using *ConSpeciFix* [30] as described above. The core genome concatenate was used to compute a distance matrix using *RAxML* version 8.2.12 (10). From these distances, the ratio of homoplasic to non-homoplasic alleles (*h/m*) was computed for i) the BSC-defined reference species alone and ii) the BSC-defined reference species + the candidate genome. Subsampling analyses were conducted as above. From this step, graphs and statistics comparing *h/m* ratios between the genomes of each BSC-defined reference species with and without the candidate genome were inferred as previous described [30]. The candidate species was inferred as a distinct BSC species when a significant and substantial reduction of gene flow was detected based on *h/m* ratios (Wilcoxon test, *P*<0.0001). When no clear reduction of gene flow was observed, the reference species and the candidate species were considered as putatively part of the same biological species and further tested for convergent mutations (see below).

### Convergent mutation test

Because our procedure is comparing various genomes, some comparisons can occasionally involve species with substantial genomic divergence. As genomes accumulate mutations during divergence, the frequency of convergent mutations increases, and this leads to the accumulation of homoplasic alleles that are the result of mutations rather than gene flow. To control for this, we simulated genome sequences for each dataset of reference + candidate species. The goal of this analysis is to generate a simulated genome sequence with similar sequence divergence and characteristics as the genome of the candidate species relative to the reference species and to estimate the ratio *h/m*_*0*_ expected to result from convergent mutations alone (Additional file 2: Fig. S3). Each sequence was evolved *in silico* with mutations but *without gene flow*. This simulated sequence was then used to estimate the ratio *h/m*_*0*_ against the reference species. The estimated values of *h/m*_*0*_ are then compared to the real *h/m* values obtained between the candidate species and the reference species (*h/m*_*cand*_). We considered cases where *h/m*_*0*_ is similar to *h/m*_*cand*_ as indicative that the signal of gene flow is actually driven by convergent mutations rather than gene flow.

First, the consensus sequence of the core genome concatenate of the BSC-defined species is generated by selecting the most frequent allele at each site. Random point mutations are then introduced *in silico* with a Jukes and Cantor model until the same sequence divergence is obtained as the one observed between the genomes of the BSC-defined species and the candidate genome. This step was conducted for each of the comparisons of BSC-redefined reference species against candidate species (13,437 comparisons). The resulting concatenate was then analyzed with the *ConSpeciFix* process as described above to infer *h/m*_*0*_ ratios. The candidate species was then considered as truly engaging in gene flow with the reference species when *h/m*_*cand*_ was found significantly higher than *h/m*_*0*_ (Wilcoxon test, *P*<0.0001).

From these metrics, we also derived the metric *h/m*_*norm*_, which quantifies gene flow between the candidate and the reference species rescaled by the amount of gene flow observed in the reference species alone (*h/m*_*ref*_), i.e., without the candidate species and by the expected amount of homoplasies introduced by convergent mutations, or, against the sequence simulated without gene flow. We expressed *h/m*_*norm*_*=* (*h/m*_*cand*_ *− h/m*_*0*_)*/*(*h/m*_*ref*_ *− h/m*_*0*_) so that *h/m*_*norm*_*=* 0 corresponds to *h/m*_*cand*_ *= h/m*_*0*_ and *h/m*_*norm*_*=* 1 corresponds to *h/m*_*cand*_ *= h/m*_*ref*_*.*

### Introgression analysis

Introgression was defined as fragments of DNA exchanged by gene flow between related species. Introgression was inferred using the concatenate of the shared core genome between each BSC-defined reference species and each candidate species (13,437 concatenates analyzed). We used a non-overlapping sliding window of 100bp to estimate the identity score of the shared core genome concatenate. For each window, the average, the minimal, and the maximal nucleotide identity were calculated for (i) the genomes of the redefined reference species alone and (ii) between the genome of the candidate species and the genomes of the reference species. We considered that a 100-bp fragment was introgressed when at least one genome of the reference species was more similar to the candidate genome than one of the other genomes of the reference species. The introgression score *S*_*i*_ was then defined as the fraction of the core genome that has been found introgressed between the candidate species and at least one genome of the reference species. Introgression scores were also computed by imposing different thresholds of sequence identity between the candidate species and the reference species for a fragment to be considered introgressed: 90%, 95%, 98%, and 100% (Additional file 2: Fig. S7). These different thresholds represent increasingly ancient introgression events. Importantly, this analysis is based on the assumption that the difference genomes of the reference species are more closely related to one another relative to the genome of the candidate species. Therefore, we did not compute the introgression score when one or more genomes of the reference species were found more related to the candidate species than to another genome of the reference species. The matrix of maximum likelihood distances (*D)* computed by *RAxML* was used to infer genome distances for each pair of candidate/reference species (see above).

### MEPS simulations

Pairs of 1Mb sequences with various levels of sequence identity (from 80 to 100%) were simulated with a Jukes and Cantor model of substitution, no indels and a GC content of 50%. For each pair of sequences, the number of potential MEPS was defined as the number of strictly identical segments of DNA shared between the two sequences. Identical fragments were identified using a scanning window of size 20bp, 30bp, 40bp, 50bp, 60bp, 70bp, 80bp, 90bp, and 100bp.

## Supplementary Information

**Additional file 1: Table S1.** Summary data of reference species.

**Additional file 2:.** Supplementary Figures S1-S8.

**Additional file 3: Table S2.** Metrics inferred for clonal species.

**Additional file 4: Table S3.** Metrics inferred for the reclassification of candidate species.

**Additional file 5: Dataset S1.** List of the reference species genomes.

**Additional file 6: Dataset S2.** List of the candidate species genomes.

**Additional file 7.** Peer review history.

## Data Availability

Genomes used in this study are listed in Dataset S1 (Additional file 5) and Dataset S2 (Additional file 6) and are freely available on *GenBank* at https://www.ncbi.nlm.nih.gov/genome/. All the core genome datasets used in this study are available at Kaggle [63–67].
