# Supplementary material for: Gene flow and introgression are pervasive forces shaping the evolution of bacterial species
Source: Genome Biol. 2022 Nov 10;23:239. doi: 10.1186/s13059-022-02809-5 (PMC9650840; doi:10.1186/s13059-022-02809-5)
Supplement: Supplementary file 2 — Additional file 2:. Supplementary Figures S1-S8. [file 13059_2022_2809_MOESM2_ESM.docx]

**Gene Flow and Introgression are Pervasive Forces Shaping the Evolution of Bacterial Species**

Awa Diop^1^, Ellis L. Torrance^1^, Caroline M. Stott^1^, Louis-Marie Bobay^1*^

^1^ Department of Biology, University of North Carolina Greensboro, Greensboro, North Carolina, 321 McIver Street, PO Box 26170, Greensboro, NC 27402 USA.

^*^Correspondence: Louis-Marie Bobay, [ljbobay@uncg.edu](mailto:ljbobay@uncg.edu)

SUPPLEMENTARY INFORMATION

Additional file 2: Supplementary Figures

Figure S1

**
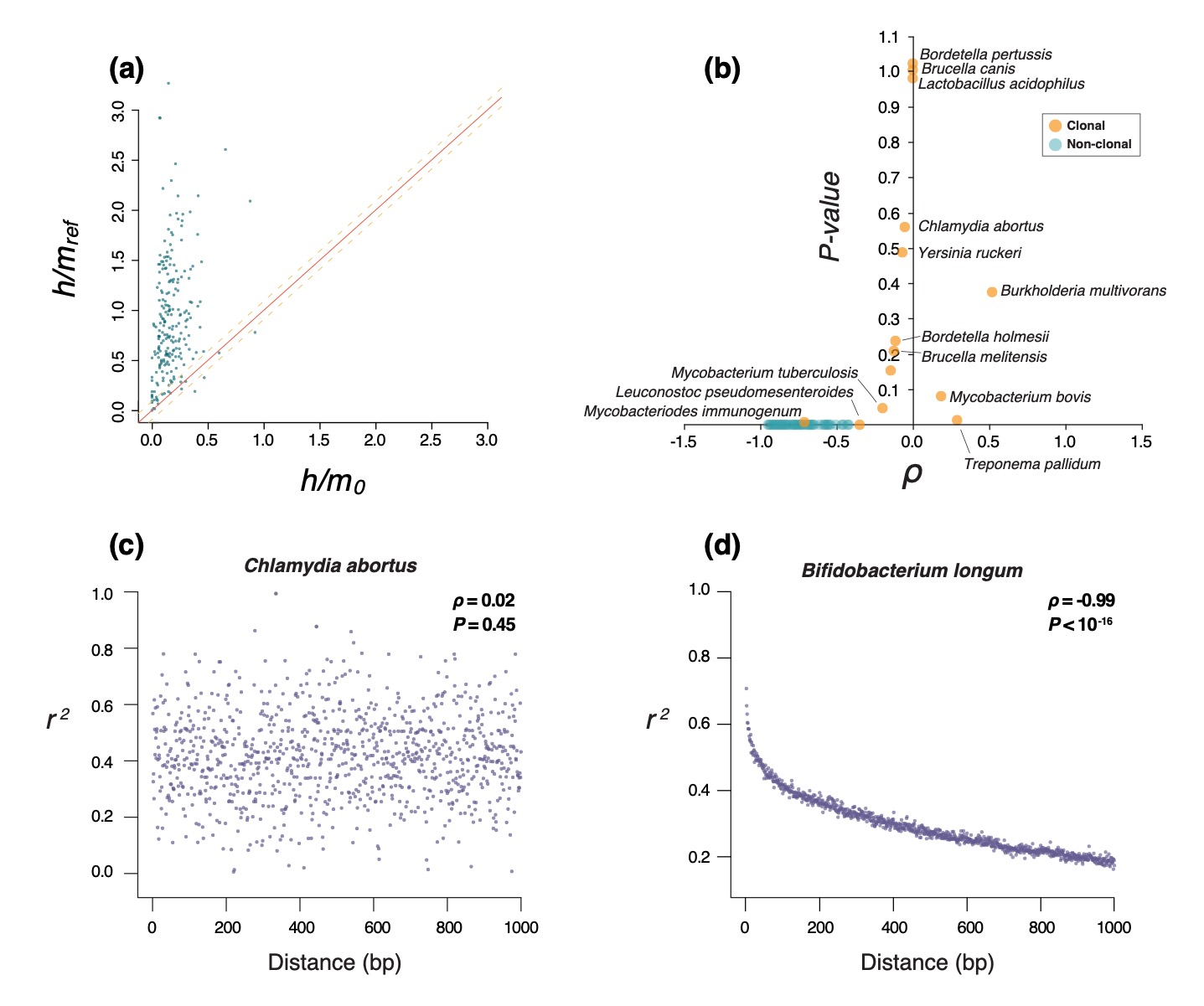
**

**Figure S1**. **Inference of clonal species.**

**a** Clonal species inferred by the simulation approach. The amount of homoplasies expected to result solely from convergent mutations was computed using simulations (*h/m_0_*). Under a scenario of clonality the amount of gene flow *h/m_ref_* inferred within each species should be very similar to *h/m_0_* (red line). The standard deviation SD of the *h/m_ref_* ratio was computed with a resampling analysis of each species. The threshold of 3.SD was selected to define clonal species (dashed lines). **b** Clonal species inferred by the Linkage disequilibrium (LD) approach. Species were considered non-clonal when a significant decrease in LD (computed with *r^2^*) relative to genomic distances between alleles was detected (*P*<0.0001, Spearman's rank correlation test) using a window of 1,000bp. For each species the Spearman's coefficient *ρ* and corresponding *P*-value is represented. Species presenting non-significant negative correlations (*P*≥0.0001) between *r^2^* and genomic distances were considered clonal (red dots). **c** Example of clonal species inferred based on LD. No significant negative correlation between *r^2^* and genomic distances was detected for *Chlamydia abortus*. **d** Example of non-clonal species inferred based on LD. A significant negative correlation between *r^2^* and genomic distances was detected for *Bifidobacterium longum.*

**Figure S2**

**
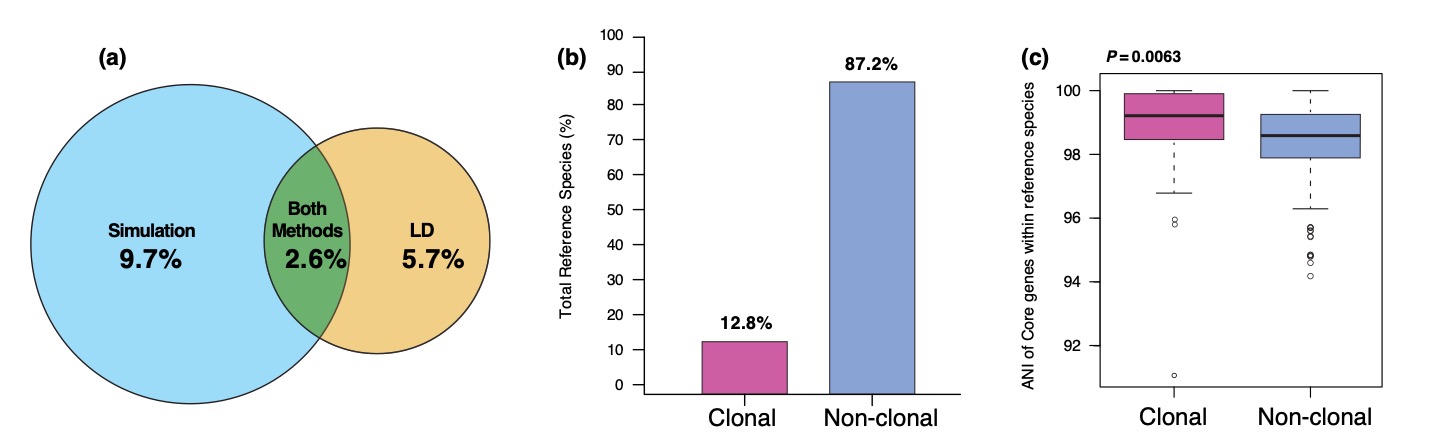
**

**Figure S2**. **Analysis of clonal species.**

**a** Percentage of clonal and non-clonal species inferred by the simulation method (blue) and by the LD method (yellow). **b** Percentage of clonal and non-clonal species inferred. Clonal species were defined here by those inferred as clonal by at least one of the two methods (simulation or LD method). **c** Distribution of average nucleotide identity of core genes values (ANI of core genes) within clonal and non-clonal species. No statistical differences were observed in ANI of core genes between clonal and non-clonal species using the Wilcoxon test.

**Figure S3**

**
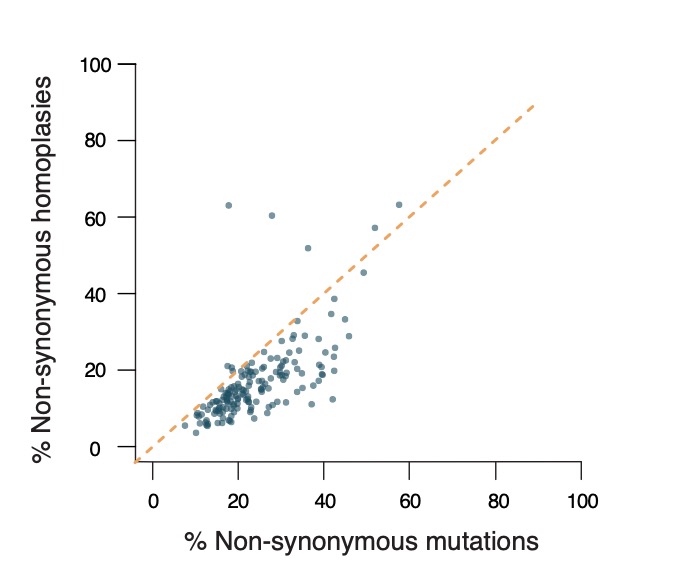
**

**Figure S3**. **Inference of synonymous and non-synonymous alleles.**

The homoplasic alleles (*h*) and non-homoplasic alleles (*m*) were classified as synonymous or non-synonymous across the core genome of all reference species. To avoid ambiguities, the analysis was focused on codons with a single polymorphic site and further restricted to codons whose polymorphic site was bi-allelic. The number of homoplasic alleles and non-homoplasic alleles found at synonymous and non-synonymous positions, respectively was then computed for each species. Species where ≥100 homoplasic alleles and ≥100 non-homoplasic alleles could be analyzed are presented.

**Figure S4**


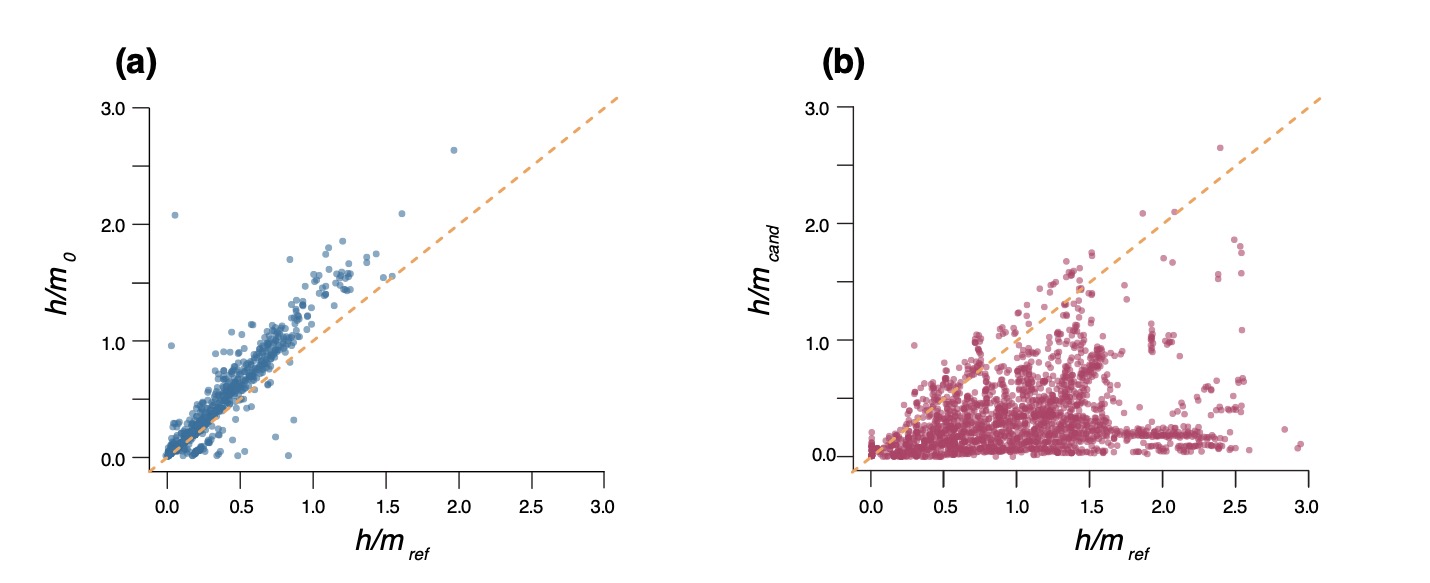


**Figure S4**. **Analysis of gene flow.**

**a** Correlation between gene flow inferred across reference species *h/m_ref_* and the amount of gene flow expected to result from convergent mutations alone *h/m_0_* based on simulations. **b** Correlation between gene flow inferred for each candidate/reference species pair (*h/m_cand_*) relative to the reference species alone *h/m_ref_*. The metric *h/m_norm_* used to identify recombining species was defined by adjusting the values of the candidate/reference species pair (*h/m_cand_*) relative to the *h/m* ratio of the reference species alone (*h/m_ref_*) and the *h/m* ratio of the simulated genomes (*h/m_0_*).

**Figure S5**

**
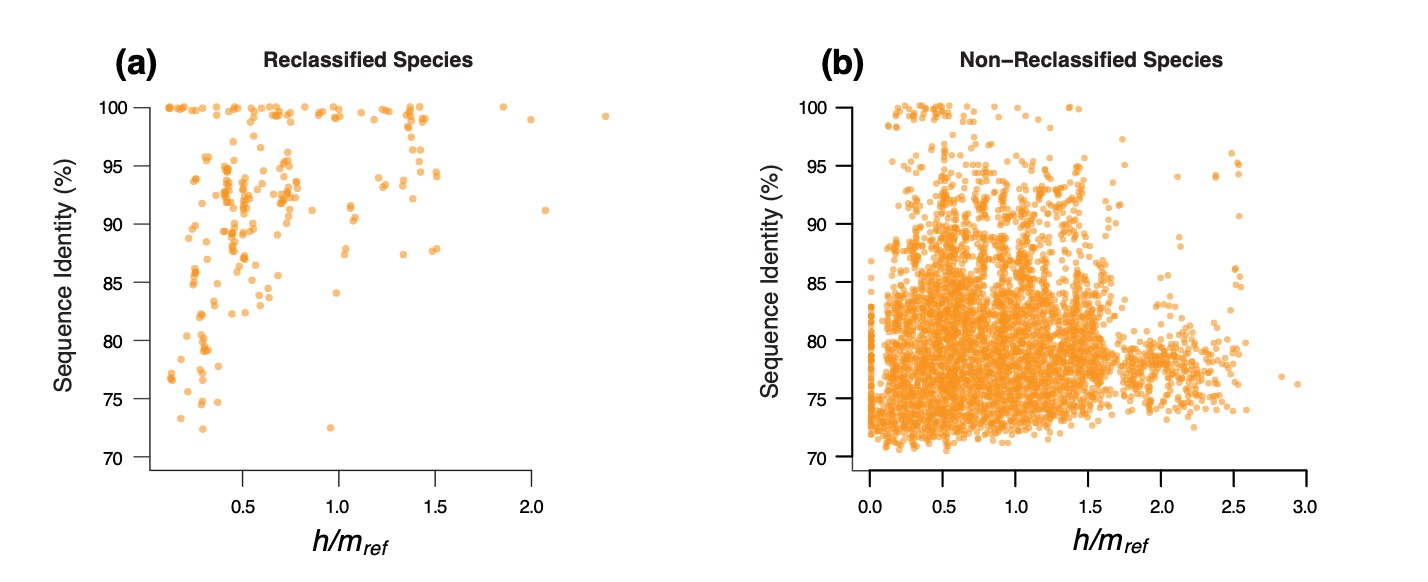
**

**Figure S5. Gene flow and sequence identity in reclassified and non-reclassified species.**

**a** Relationship between maximum sequence identity (ANI of core genes) and *h/m* ratio of the reference species alone (*h/m_ref_*) for the pairs of candidate/reference species that were reclassified into a single species. **b** Relationship between maximum sequence identity (ANI of core genes) and *h/m* ratio of the reference species alone (*h/m_ref_*) for the pairs of candidate/reference species that were not reclassified into a single species.

**Figure S6**


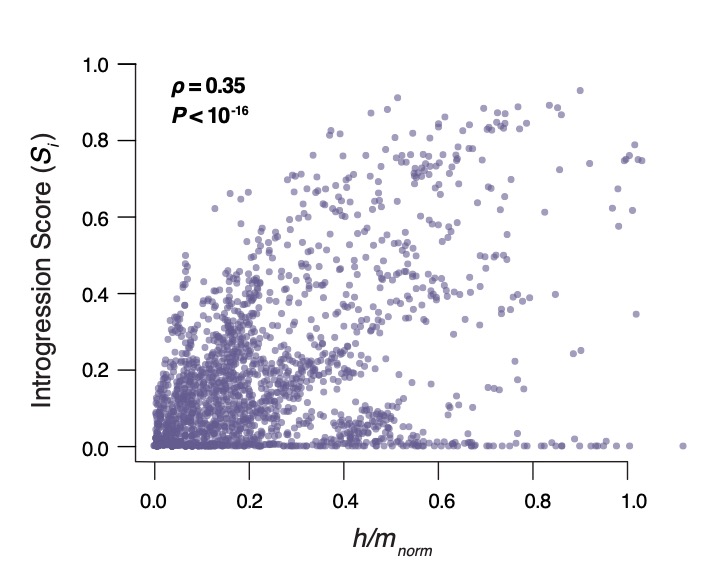


**Figure S6**. **Relationship between introgression score and *h/m* ratio.**

Introgression score *S_i_* was computed for each pair of candidate/reference species. *S_i_* represents the fraction of the core genome inferred as introgressed between the reference and the candidate species. The metric *h/m_norm_* represents the ratio of homoplasic to non-homoplasic alleles inferred between the candidate and the reference species. The ratio *h/m_norm_* is adjusted by the amount of homoplasies expected to result from convergent mutations (*h/m_0_*) as inferred by simulations and adjusted by the amount of gene flow inferred in the reference species alone (*h/m_ref_*).

**Figure S7**

**Figure S7. levels of introgression across lineages (Class level).**

**a** Maximal introgression scores (defined at ≥95% sequence identity) inferred in the core genome between candidate/reference species for each class (averaged across species). **b** Average introgression scores (defined at ≥95% sequence identity) inferred in the core genome between candidate/reference species for each class (averaged across species). **c** Maximal introgression scores (defined at 100% sequence identity) between candidate/reference species in each class (averaged across species). **d** Average introgression scores (defined at 100% sequence identity) between candidate/reference species in each class (averaged across species). The number shown above each graph represents the number of reference species within each class.

**Figure S8**


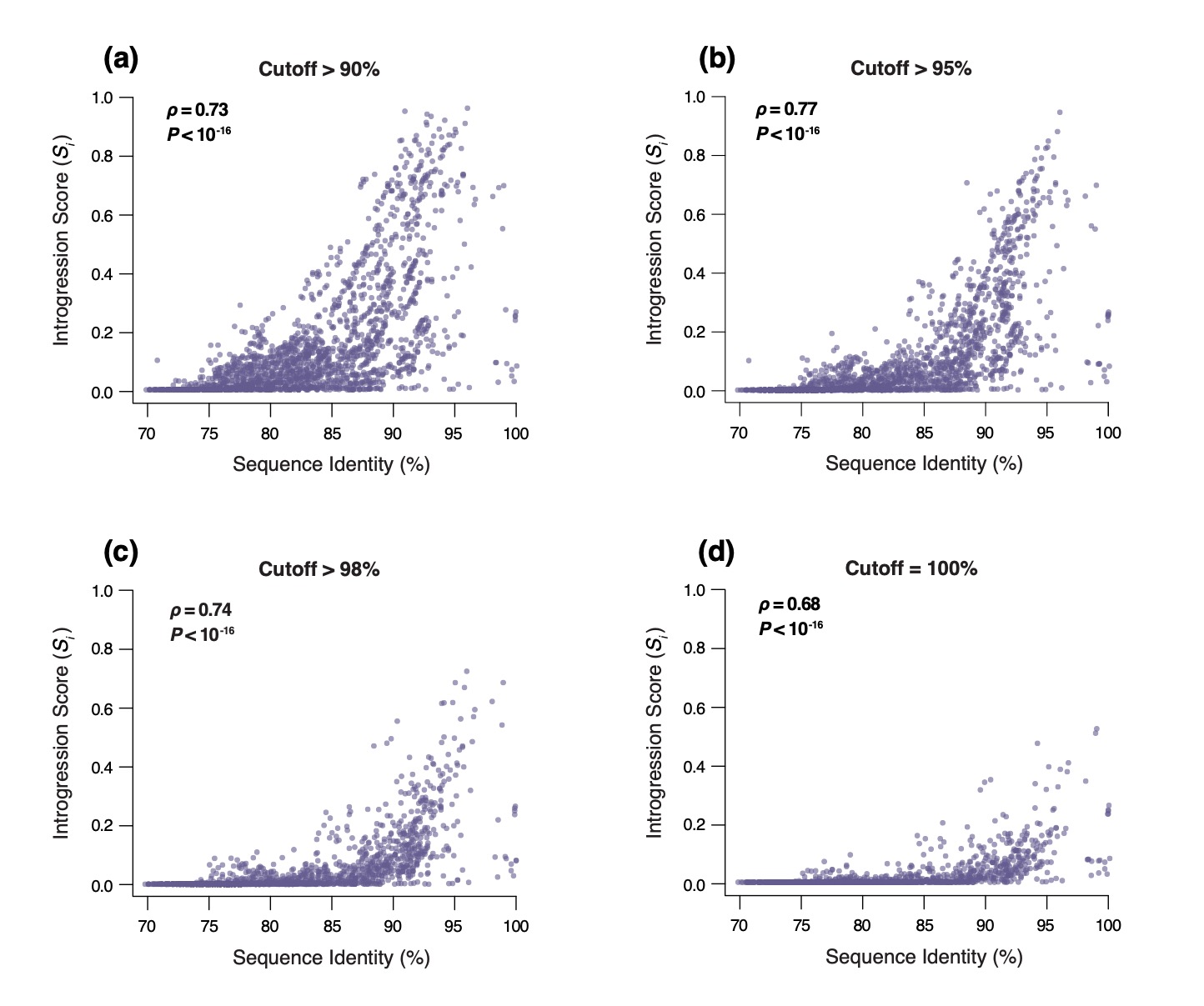


**Figure S8**. **Inference of introgression using different sequence identity thresholds.**

Each graph represents the introgression scores (*S_i_*) between candidate and reference species that were not reclassified as part of the same species. *S_i_* represents the fraction of the core genome that shows evidence of introgression between the candidate species and at least one genome of the reference species. Introgressed regions were defined as 100bp fragments that were more similar to the candidate species than at least one of the genomes of the reference species. Coefficient correlation *ρ* and *P*-value were estimated with Spearman's rank correlation test. **a** Only introgressed fragments with ≥90% sequence identity between the candidate and reference species were considered as introgressed **b** Only introgressed fragments with ≥95% sequence identity between the candidate and reference species were considered as introgressed. **c** Only introgressed fragments with ≥98% sequence identity between the candidate and reference species were considered as introgressed. **d** Only introgressed fragments with 100% sequence identity between the candidate and reference species were considered as introgressed.
