# Supplementary material for: Gene flow and introgression are pervasive forces shaping the evolution of bacterial species
Source: Genome Biol. 2022 Nov 10;23:239. doi: 10.1186/s13059-022-02809-5 (PMC9650840; doi:10.1186/s13059-022-02809-5)
Supplement: Supplementary file 7 — Additional file 7. Peer review history. [file 13059_2022_2809_MOESM7_ESM.docx]

**Review history**

**First round of review**

**Reviewer 1**

The article by Diop et al. attempted to quantify recombination of core genes in order to define species based on sexual reproduction (introgression). The main approach is to identify mutations (SNPs) between closely related genomes and designate them as SNPs shared by recombination (i.e., exchange of genetic material) or homoplacies; that is, the mutation is raised independently in multiple genomes by chance alone. While I agree with the authors that homoplacies are likely rare enough, e.g., to get the same exact mutation in two genomes/population in a 100bp window independently and by chance alone has a very low likelihood (e.g., 1 in 100 substitutions, plus it has to be the same nucleotide from the 3 possibilities compared to the ancestor nucleotide), it represents a major limitation of the approach that it does not account for selection, in the opinion of this reviewer. For instance, I often see in mapping metagenomic reads against a reference genome a single SNP (relative to the reference genome) that is shared by many reads over a stretch of an otherwise identical 100bp windows. The authors would consider these a major recombination event by their approach that would overshadow mutation in the specific window of my example, but it could as well be simply a strong selection for the specific nucleotide substitution. Recall also here the work of Rich Lenksi and colleagues where many (most?) of the fixed mutations in his laboratory E. coli strains originate from selection from the growth media and are precisely shared among independent lineages (cultures). Without taking selection into account, or disprove it, one cannot really assess the value of the results reported here (and in the previous papers by the senior author, I have to say). In my view, it is not possible to account for the effect of selection on shared mutations (e.g., we do not have appropriate data for this from natural populations; Lenki's cell lines is a different case) or the authors have not provided an approach to deal with its effects. So, selection is likely a very important factor that can not be easily accounted for, but it is completely disregarded here.
Further, in order for introgression to serve as a force of population (or species) cohesion, it has to occur randomly across the genome, not only in a few, selected genes of the genome; the latter is just horizontal gene transfer, driven by selection (which we know by now that almost all bacteria do/undergo). One of the examples provided by the authors, the Campylobacter coli vs. Camplylobacter jejuni has been shown to represent a case of high r/m but driven by selection for specific functions and thus, it is highly unlikely to lead to species convergence unlike what is suggested by the authors (see Caro-Quintero et al, J. Bacterilogy, 2009 and subsequent publications such as Lefébure et al., Genome Biology 2010; Snipen et al., Microb Informatics Exp, 2021). The authors have not shown that the recombined fragments are randomly found across the genome, as is the case for sexual eukaryotes. Moreover, the effect of ecological differentiation in gene flow is not discussed at all, but it could be a major underlying reason for disruption (or not) of the gene flow, as the C. jejuni-C. coli example also indicates (these species have overlapping niches which account -at least in part- for their genetic exchange; yet, they are unlikely to converge into one species as I wrote above and previous literature indicates). Finally, the authors seem to be missing some key literature on the topic. Please see also specific comments below about the latter two points.

Minor/Specific points.
Line 21-22. I think this statement is too general, vague. Something more specific, e.g., about what is the force of species cohesion?

Line 29. 95% ANI is not a strict threshold but an "average" property that emerges from genome comparisons; it should be adapted depending on the age of the populations evaluated; e.g., younger populations emerging from a recent population diversity sweep event tend to show a higher ANI boundary. See Rodriguez-R et al., Nat. Comm. 2021 for more details and discussion (cited by the authors). It is interesting that the 95% ANI is somewhere in the middle of the range provided here, consistent with previous literature and conclusions, but unlike the "feeling" that the abstract leaves (that is, that 95% ANI does not work; the opposite seems to be the case if one considers the Rodriguez-R et al study and its recommendations about ANI)…
Further, and perhaps more importantly, what is the effect of ecological differentiation in the gene flow (e.g., genomes that are found in different ecological niches are unlikely to exchange genes because they are not in close enough proximity)? Maybe ecological differentiation is important, if not the dominant underlying reason?

Line 30. This reads to me like the fragmented speciation hypothesis (Retchless and Lawrence, PNAS, 2010), which has not been tested true as far as I am aware (it is mostly tested false, I believe). The issue is that any genome pair in the >90% ANI range will have some identical fragments shared. So, it is not clear why this is attributed to exclusively to recombination. Related to the latter, Figure 3C shows with the blue line the number of fragments one would expect by chance alone, without recombination (simulated data with clonal evolution), I think (but I am not sure, because the figure legend is not clear about this), the authors use it to show that several genome pairs have more identical windows than expected by clonal reproduction alone i.e., these pairs are found to the left of the blue line. If this is indeed the case, then how the blue line was exactly drawn (e.g., how the underlying data were produced) and, perhaps more importantly, why there would be genome pairs (real genomes) to the right of the line? That is, genome pairs that have less identical fragments than expected by mutation alone? This does not make much sense to me, and it worth a discussion, at least.

Line 32. I am confused by this statement; are you talking about recombination (introgression) within species here or across species? Because the word "boundary" in the same sentence implies across species. Please clarify and be more precise in your text.

Line 62. Are you using "introgression" across species or also within? (species defined by some arbitrary identity threshold). Not clear as written.

Line 85-86. So, it is ANI of core genes, right? Why not write it like this? CNI is not used much...

Line 92. Why not selection for specific mutations? This has been a big issue of the senior author's work not only here but in his previous publications. Without taking selection into account, or disprove it, one cannot really assess the value of the results reported here and previously. I am aware that the senior author's previous publications discussed selection somewhat (although I think, at the end, they decided to neglect it?) but a brief discussion should be included here for the shake of completeness; as is the case for the effect of ecology on gene flow.

Line 98-100. Again, it appears that the simulation modeling assumes no selection, which is most likely an unrealistic assumption for real (not-simulated), natural populations.

Line 159. You mean core ANI or whole-genome ANI; core-ANI will be a little higher than whole ANI (so, ~96% ANI as opposed to ~94% mentioned elsewhere in the paper would be the threshold from the literature based on core ANI). Please specify for readers can compare results to previous ANI studies.

Line 191-194. Maybe some core genes are exchanged but to have sexual reproduction, these genes need to be randomly around the genome. The C. jejuni and C. coli is a case of biased selection for genes related to antibiotic usage and their ecology; it can not lead to convergence of the two distinct species. See Caro-Quintero et al, J. bac 2009. This is exactly a great example of what I am talking about on the role of selection as well as ecological differentiation (or lack of it).

Line 200. Lack of strong selection by varying environmental conditions could be the explanation why symbiotic species and Mycobacterium species do not show signal of differentially shared mutations/SNPs (attributed to recombination by the authors), as compared to free-living organism that often undergo variable selection conditions due to environmental changes, including seasonal changes (e.g., inside a host is a more stable environment, by comparison, likely).

Line 213-214. Please see the fragmented speciation model in PNAS. The authors are apparently missing some key literature on the topic.

Line 292. How this method differs from more traditional ANI e.g., using the FastANI tool (Jain et al, Nat. Comm. 2018)? Will be nice to include so readers can make direct comparisons.

**Reviewer 2**

Diop et al present a comprehensive analysis of recombination rates in bacteria. They use a suite of methods to infer the frequency of recombination in different bacterial species, and they ultimately show that the recombination can occur across a wide range of nucleotide identities (90-98%). Remarkably, they also find evidence of "introgression" across much greater genetic distances, indicating that recombination-driven gene flow may occur even across fairly divergent lineages.

It's difficult to overstate the importance of this work given the critical role of recombination in bacterial evolution coupled with the difficulty microbiologists have had in defining species based on gene flow. The authors also put forward a convincing argument for how to define "natural" species boundaries in bacteria using core concepts from the biological species concept (BSC) that both microbiologists and evolutionary biologists will find appealing. Most species definitions have been based on quasi-arbitrary cutoffs that are naturally quite simplistic (Jain et al, Nature Communications, 2018). Most everyone recognized that these one-size-fits-all cutoffs are not appropriate in all situations, but given the daunting challenge of evaluating recombination rates across the TOL it has seemed reasonable to use this as an estimate. Diop et al present detailed analyses that reshape these perspectives and provide much-needed quantitative information on recombination rates, natural species boundaries, and even the fascinating but underappreciated role of introgression. This work will be an important foundation for future studies and will be of great interest to microbiologists and evolutionary biologists alike. I predict it will be highly cited.

I have no major concerns or revisions, but I do have some suggestions for clarifications or possible additional text inclusion below:

-This work has important implications for pan-genomics. Pangenomics has become a subject of intense debate recently (see https://doi.org/10.1038/nmicrobiol.2017.40 and associated discussion) but it isn't even clear how to define pangenomes using current methods. Usually researchers rely on the 95% identity threshold or other metrics not based on gene flow, but as this work shows, these cutoff can lead to species-clusters that include distinct populations with no gene flow (and inflate pangenome sizes) or fail to capture the full diversity of a pangenome when recombination occurs across a greater genetic distance. This is just a suggestion, but if the authors do not feel that it detracts from the overall message of their study too much it may be worth including a few sentences outlining the implications of their findings for defining pangenomes more rigorously. In the future it will be important to use the BSC-defined groups here to explore patterns of pangenomic variability, and see if this changes any previous conclusions in this area.

-The framework used here for identifying h/m ratios is quite impressive, especially regarding how the simulations were done to identify expected patterns for clonality for each species. This is a nontrivial task. I understand this workflow may not easily fit into a user-friendly pipeline, but in the future I encourage the authors to find a way to make their methods a bit more accessible so that others can easily define BSC-clusters from their own input genomes. Some of these methods are already available (e.g. CoreCruncher) so it's possible the authors are already doing this, but I just wanted to stress the importance of accessible approaches to calculating h/m ratios and introgression given their broad implications for species definitions and pangenomics. Many researchers simply won't be able to perform the multiple steps, especially the simulations, on their own.

-is it possible some homoplasic alleles derive from low-complexity regions, rather than true recombination events? Or perhaps the simulations would account for this?

-Line 111- awkward wording- suggest change to "not all endosymbiont species were found…"

-Line 118-120- I'm not sure I agree with the logic that endosymbionts are essential to their host and therefore escape extinction, because many endosymbionts do go extinct and are replaced (https://doi.org/10.1038/s41396-017-0024-6). Perhaps clonality coupled with population bottlenecks is actually a recipe for extinction, and these endosymbionts just haven't gotten there yet?

-Line 133- where does 2446 come from? I understood after checking the methods, but perhaps a short description of where these genomes come from would help improve the logical flow here.

-Line 156- add cites for the E. coli/Shigella issue

-Line 181- "more similar" - what about cases where all comparisons are 100 percent identical? Would that not also represent very-recent introgression?

-Line 187-189- this is a very important statistic but it could be worded more clearly. Can it be re-worded so that authors get a more natural impression of the scale of introgression? Perhaps the percent of species that had introgression levels exceeding 1 percent of their genomes? And Figure S6 seems to be quite important for making the point that introgression is pervasive- perhaps this should be in the main text?

-Something about the taxonomic scale of introgression may be useful for readers (i.e., cross-genus or cross-family?), even if taxonomy is a mess and encompasses different levels of diversity across groups.

-The discussion of MEPS is quite fascinating and adds an important mechanistic element to this study. This is key because it elevates the work compared to other purely data-driven studies on the subject that have promoted the 95% ID threshold. It shows the authors have thought quite deeply about this subject from a microbial standpoint.

-Figure 3C is quite striking. For par C, how can a genome have an introgression score near 50% and a percent identity >95% ID but still be considered part of a different BSC-species? It seems like this is a huge amount of gene flow between quite similar genomes, so it would constitute enough recombination to permit classification within the same species. Is this because the h/m was still not higher than one would expect due to simulations? I suppose closely-related but distinct species may still have high nucleotide identity, but without comparing the h/m and Si it isn't possible to tell whether this similarity is because of introgression between species or simply similarities that persist due to recent shared ancestry between separate species?

-I enjoyed the discussion on individual species that had notable levels of gene flow and introgression. Including a few more examples throughout the text would be welcome- I found myself wondering if there was any clear link between species that experienced introgression, or species that recombined across the largest genetic distances. Of course all this information can be looked up in the supplement, but a bit more detail in the text might be nice. This is a concise manuscript so I think there should be plenty of room.

-Line 259 - "will be compared" - seems awkward to use future tense here.

-Line 265 "your list" - awkward wording

-The methods are very detailed.

-line 282- could there be any systematic bias introduced between muscle-aligned and mafft-aligned species? I only ask because I have noticed differences in the alignments provided by these tools,
though this was mainly an issue for divergent sequences.

**Reviewer 3**

The authors present a very interesting and well-conceived analysis in order to better quantify the extent to which gene flow shapes bacterial genomes. Importantly, this analysis is not limited to a small subset of taxa, but rather spans most of the known bacterial taxonomy and thus represents a significant advance in the fields of microbial evolution and bacterial speciation. I think it would be of great interest to biologists in a more general sense, as it provides further evidence that evolutionary theory built for sexually-reproducing organisms can be broadly applied to bacteria.

That said, I have a few concerns and questions regarding the authors' methodology.

1) From my reading, I'm not sure that the lead statistic that 10% of analyzed species are truly clonal is fully justified. The authors use a leave-one-out approach to account for uncertainty in the observed h/m ratio, but this does not account for uncertainty in the "expected" h/m ratio generated from simulations. As I understand it, only one simulation for each species (the simulation with the closest CNI to the full dataset) was chosen to get a point estimate of the expected h/m ratio. How much variance is there in the h/m ratio for a less restricted subset of the simulations and can this be used to derive some empirical confidence intervals for the fraction of analyzed species that are truly clonal?

2) Related to point 1, how do the authors explain cases where the observed h/m is significantly lower than the simulated h/m? (E.g., supplementary table 2, Xanthomonas_euvesicatoria)? Is it possible to be "more clonal" than expected, or is there something that the simulations are not fully capturing about clonal evolution?

3) Much of the analysis here depends on the definition of the core genome, which in turn depends on species definitions to begin with. To what extent are h/m estimates from the core genome of a species that has a CNI of ~98% comparable to the h/m estimates for species with a CNI of say, 90%? I don't have a particularly compelling suggestion here, but I think this point deserves more discussion.

4) Why was only one genome from a candidate species used when defining the core genome of each candidate/reference species pair? In the case where CNI within a candidate species was relatively low, wouldn't one expect that the specific representative chosen would have a large effect on the inference of the core genome for the candidate/reference pair?

5) In Figure 3C, is there an explanation for why the observed recent introgression score is actually lower than expected at high identity?

Minor points:

1) There are a few instances where it seems a term is defined after it is first used or should be used. For example, in line 131, does "every species" mean "every candidate species"? And in lines 140-144, h/m0 is introduced a little before it is described.

2) It was initially unclear to me that the introgression analysis was only conducted on candidate species that did not get assigned to a BSC reference species.

3) Figure 3C: Is the y-coordinate of the red line meant to be the MEPS frequency and not the introgression score?

**Authors’ response to reviewers**

**Reviewer #1:**

Major points:

The article by Diop et al. attempted to quantify recombination of core genes in order to define species based on sexual reproduction (introgression). The main approach is to identify mutations (SNPs) between closely related genomes and designate them as SNPs shared by recombination (i.e., exchange of genetic material) or homoplasies; that is, the mutation is raised independently in multiple genomes by chance alone. While I agree with the authors that homoplasies are likely rare enough, e.g., to get the same exact mutation in two genomes/population in a 100bp window independently and by chance alone has a very low likelihood (e.g., 1 in 100 substitutions, plus it has to be the same nucleotide from the 3 possibilities compared to the ancestor nucleotide), it represents a major limitation of the approach that it does not account for selection, in the opinion of this reviewer. For instance, I often see in mapping metagenomic reads against a reference genome a single SNP (relative to the reference genome) that is shared by many reads over a stretch of an otherwise identical 100bp windows. The authors would consider these a major recombination event by their approach that would overshadow mutation in the specific window of my example, but it could as well be simply a strong selection for the specific nucleotide substitution. Recall also here the work of Rich Lenksi and colleagues where many (most?) of the fixed mutations in his laboratory E. coli strains originate from selection from the growth media and are precisely shared among independent lineages (cultures). Without taking selection into account, or disprove it, one cannot really assess the value of the results reported here (and in the previous papers by the senior author, I have to say). In my view, it is not possible to account for the effect of selection on shared mutations (e.g., we do not have appropriate data for this from natural populations; Lenki's cell lines is a different case) or the authors have not provided an approach to deal with its effects. So, selection is likely a very important factor that cannot be easily accounted for, but it is completely disregarded here.

***We thank the Reviewer for their input. We do not share the view of the Reviewer that selection can have a significant impact on the levels of homoplasies.***

***As stated by the Reviewer, convergent mutations due to selection are expected to be rare and our analyses typically include thousands of homoplasies for each species. This would require selection to act on a tremendous number of alleles.***

***The idea behind selection driving convergent mutations (and the studies cited) is based on the assumption of adaptive evolution/selection, not purifying selection. It should be noted that the studies cited by the Reviewer are cases of bacteria put under very strong selective pressures (e.g. antibiotic resistance) and in very simplified environments and the cell lines of Lenski are clearly maladapted to their environment. In contrast, the homoplasies in our study have been detected in the core genome, where genes are evolving under purifying selection. Adaptive evolution is not expected to be strong or common in these genes. It is also very unlikely that adaptation would occur through similar independent mutations in natural conditions where selective pressures are complex and multiple. Bacterial strains have different genotypes and gene content, which implies that the same mutation is unlikely to provide the same adaptive advantage in different genomic backgrounds.***

***Nevertheless, the point raised by the Reviewer can be tested with our dataset. It is expected that adaptive convergence would affect non-synonymous sites rather than synonymous ones. Although synonymous sites can experience some selection, those are much weaker and are not the primary target of adaptive evolution. In addition, selective pressures acting on synonymous sites are typically associated with codon usage and/or nucleotide composition of an entire gene overall. This last point indicates that selection at synonymous positions is not expected to affect a particular site, but, rather, the overall sequence of the gene.***

***We tested whether the homoplasies inferred in our dataset were predominantly observed at synonymous or non-synonymous sites, and we did the same for non-homoplasic alleles. If selection were driving the accumulation of homoplasies, those would be strongly biased toward non-synonymous sites. We classified all homoplasic and non-homoplasic alleles in our dataset as synonymous or non-synonymous. To avoid any possible ambiguity, we focused our analysis on codons with a single polymorphic (bi-allelic) site. Our results revealed that for the vast majority of species (>95%), non-homoplasic alleles are more frequently non-synonymous than homoplasic alleles (see Figure S3). These results further show that over 50% of the homoplasies detected in a species are typically observed at synonymous sites. As mentioned above, synonymous sites are evolving under quasi-neutral evolution and the weak selective pressures acting on these sites are typically related to codon usage and gene expression. Although it is possible that some homoplasic alleles may be the result of adaptive evolution, our results clearly show that this not impacting a substantial fraction of sites since the bias is even weaker for homoplasic alleles than non-homoplasic alleles.***

***We have included these results in the revised manuscript lines 116-134, Figure S3 and lines 444-455 of the Methods section.***

Further, in order for introgression to serve as a force of population (or species) cohesion, it has to occur randomly across the genome, not only in a few, selected genes of the genome; the latter is just horizontal gene transfer, driven by selection (which we know by now that almost all bacteria do/undergo). One of the examples provided by the authors, the Campylobacter coli vs. Camplylobacter jejuni has been shown to represent a case of high r/m but driven by selection for specific functions and thus, it is highly unlikely to lead to species convergence unlike what is suggested by the authors (see Caro-Quintero et al, J. Bacterilogy, 2009 and subsequent publications such as Lefébure et al., Genome Biology 2010; Snipen et al., Microb Informatics Exp, 2021). The authors have not shown that the recombined fragments are randomly found across the genome, as is the case for sexual eukaryotes.

***We apologize if our point wasn't clear, but we did not mean to insinuate that introgression is expected to lead to species convergence. We were simply citing the conclusions reached by the authors of the cited* Campylobacter *study (Sheppard et al.). We do agree with the Reviewer that introgression is unlikely to drive species fusion and that is not the point we were trying to make. We simply used the term "introgression" as gene flow between the core genomes of distinct species. We have now removed this statement from the manuscript.***

Moreover, the effect of ecological differentiation in gene flow is not discussed at all, but it could be a major underlying reason for disruption (or not) of the gene flow, as the C. jejuni-C. coli example also indicates (these species have overlapping niches which account -at least in part- for their genetic exchange; yet, they are unlikely to converge into one species as I wrote above and previous literature indicates).

***We agree that ecological differentiation is likely a major force affecting gene flow. We were not trying to imply that ecology is not a significant factor, but we simply did not expand much on this point because this was not the focus of our study. Our results suggest that sequence divergence re-enforces population differentiation, but we do not pretend to explain what forces are originally driving differentiation and reductions in gene flow. We have clarified this point in the manuscript (lines 240-242).***

Finally, the authors seem to be missing some key literature on the topic. Please see also specific comments below about the latter two points.

***There are many works to cite on this topic. We are now citing additional works relevant for our study (please see citations in lines 117, 124, 164 187, 235, 239, 242 and 286 in the revised manuscript for instance). We thank the Reviewer for bringing several of these studies to our attention*.**     

**Minor/Specific points.**
Line 21-22. I think this statement is too general, vague. Something more specific, e.g., about what is the force of species cohesion?
***The sentence has been clarified (please see line 22 in the revised manuscript).***

Line 29. 95% ANI is not a strict threshold but an "average" property that emerges from genome comparisons; it should be adapted depending on the age of the populations evaluated, e.g., younger populations emerging from a recent population diversity sweep event tend to show a higher ANI boundary. See Rodriguez-R et al., Nat. Comm. 2021 for more details and discussion (cited by the authors). It is interesting that the 95% ANI is somewhere in the middle of the range provided here, consistent with previous literature and conclusions, but unlike the "feeling" that the abstract leaves (that is, that 95% ANI does not work; the opposite seems to be the case if one considers the Rodriguez-R et al study and its recommendations about ANI)…

***We agree with the Reviewer and we have now rephrased this statement. Note that we did not want to imply that the ~95% ANI threshold is an absurdity. This threshold (as shown by the studies cited by the Reviewer) has been supported by multiple works and many empirical observations. We believe that our results provide some theoretical foundations for why this threshold is a good approximation (although the exact number to use across lineages remains an issue). As stated by the Reviewer, the 95% ANI threshold is somewhere in the middle of the range of species boundaries that we identified - and we thought that this was a very exciting result - but perhaps we did not emphasize this point enough. We have now modified the manuscript to underline this conclusion more explicitly in the Abstract and in the Conclusion sections in the revised manuscript (please see lines 29-30 and 296-300).***

Further, and perhaps more importantly, what is the effect of ecological differentiation in the gene flow (e.g., genomes that are found in different ecological niches are unlikely to exchange genes because they are not in close enough proximity)? Maybe ecological differentiation is important, if not the dominant underlying reason?

***We agree with this comment. We did not want to imply that ecology is not a key factor for speciation. We are now discussing this point in more details in the Conclusion section (please see lines 293-294 and 304-305).***

Line 30. This reads to me like the fragmented speciation hypothesis (Retchless and Lawrence, PNAS, 2010), which has not been tested true as far as I am aware (it is mostly tested false, I believe).

***Some of our conclusions are indeed related to the fragmented speciation hypothesis. However, there are some main differences. For instance, our model does not assume that entire genomic regions would become sexually isolated and expand to neighboring regions. Our results do not support this idea either. We must also emphasize that some aspects of the fragmented speciation hypothesis appeared somewhat unclear to us, and we did not want to extrapolate what the original authors had in mind. We are now discussing this point in the revised manuscript (please see lines 285-288).***

The issue is that any genome pair in the >90% ANI range will have some identical fragments shared. So, it is not clear why this is attributed to exclusively to recombination. Related to the latter, Figure 3C shows with the blue line the number of fragments one would expect by chance alone, without recombination (simulated data with clonal evolution), I think (but I am not sure, because the figure legend is not clear about this), the authors use it to show that several genome pairs have more identical windows than expected by clonal reproduction alone i.e., these pairs are found to the left of the blue line. If this is indeed the case, then how the blue line was exactly drawn (e.g., how the underlying data were produced) and, perhaps more importantly, why there would be genome pairs (real genomes) to the right of the line? That is, genome pairs that have less identical fragments than expected by mutation alone? This does not make much sense to me, and it worth a discussion, at least.  
***Very few identical fragments are expected to result from vertical inheritance. Indeed, our analysis does not simply consist of identifying identical regions between species. We are identifying fragments that are more similar to other species than across conspecific strains. We are also ensuring that the conspecific strains are overall more related to each other than they are to the external species.***

***The blue line was simply drawn as the expected number of 100bp identical fragments between two genomes of various divergence rates (which is possibly what is introducing the confusion here). This was a simple way for us to indicate the upper limit of how much introgression could be inferred by chance, but in reality, we expect this level to be much lower than that. Indeed, for this to happen through vertical evolution, one would not only need to find a 100bp fragment identical across a strain of the reference species and the candidate species, but this fragment would also need to be different across the strains of the reference species. For this reason, it is clearly expected to find genome pairs on the right side of the blue line. The exact number of identical fragments expected to result from vertical evolution is very small but difficult to predict since it is lineage-specific (it depends on the divergence between species and on the levels of polymorphisms within each species). We realize that these explanations were not made clear enough in the manuscript and we have now provided more details about this (please see lines 249-259 in the revised manuscript).***

Line 32. I am confused by this statement; are you talking about recombination (introgression) within species here or across species? Because the word "boundary" in the same sentence implies across species. Please clarify and be more precise in your text.

***This sentence refers to "between species". We have defined introgression as "gene flow between species". We have clarified our terminology throughout the text.***

Line 62. Are you using "introgression" across species or also within? (species defined by some arbitrary identity threshold). Not clear as written.
***We apologize for the confusion; "introgression" always refers to gene flow across species in the manuscript. This has been clarified throughout the manuscript.***

Line 85-86. So, it is ANI of core genes, right? Why not write it like this? CNI is not used much...

***We have now referred "CNI" as "ANI of core genes" to follow the Reviewer's recommendation (please see lines 86-87 in the revised manuscript).***

Line 92. Why not selection for specific mutations? This has been a big issue of the senior author's work not only here but in his previous publications. Without taking selection into account, or disprove it, one cannot really assess the value of the results reported here and previously. I am aware that the senior author's previous publications discussed selection somewhat (although I think, at the end, they decided to neglect it?) but a brief discussion should be included here for the shake of completeness as is the case for the effect of ecology on gene flow.

***As stated above, we do not share the opinion of the Reviewer that adaptive evolution could lead to a significant amount of convergent alleles. We are now presenting new results that do not support a substantial impact of selection on the emergence of homoplasies. We are discussing this point in the Results / Discussion section and added Figure S3 (please see lines 116-134 and 444-455 in the revised manuscript).***

Line 98-100. Again, it appears that the simulation modeling assumes no selection, which is most likely an unrealistic assumption for real (not simulated), natural populations.

***This is not totally accurate. Our simulations do include a model of selection. Based on the levels polymorphisms observed across codon positions in each species' core genome, we simulate genome evolution with three different rates of substitution across the three codon positions. It is true however that we did not attempt to model positive selection for the reasons explained above and this is now discussed in the revised manuscript (please see lines 108-111).***

Line 159. You mean core ANI or whole-genome ANI; core-ANI will be a little higher than whole ANI (so, ~96% ANI as opposed to ~94% mentioned elsewhere in the paper would be the threshold from the literature based on core ANI). Please specify for readers can compare results to previous ANI studies.

***The sentence has been clarified in the revised manuscript in lines 191-192.***

Line 191-194. Maybe some core genes are exchanged but to have sexual reproduction, these genes need to be randomly around the genome. The C. jejuni and C. coli is a case of biased selection for genes related to antibiotic usage and their ecology; it cannot lead to convergence of the two distinct species. See Caro-Quintero et al, J. bac 2009. This is exactly a great example of what I am talking about on the role of selection as well as ecological differentiation (or lack of it).

***Introgression patterns are not necessarily random since selective pressures can act on introgressed DNA. Although the original events of DNA exchanges are most likely random, many introgressed loci are expected to be lost over time due to recombination with conspecifics. Some introgressed segments can be retained in a non-random fashion thanks to selection. A famous example of this process has been observed in humans, where Tibetan populations were found to possess introgressed DNA from Denisovans and this is associated with a signal of selection due to the advantage that this locus confers to populations living in high altitude (Huerta-Sánchez et al., Nature 2014).***

***As stated above, we do not claim that introgression can lead to the full convergence of two distinct species. This is not the point of our analysis and our results do not provide any support for or against this hypothesis. We apologize if we unintentionally conveyed this idea. We have now clarified the text throughout the manuscript.***

Line 200. Lack of strong selection by varying environmental conditions could be the explanation why symbiotic species and Mycobacterium species do not show signal of differentially shared mutations/SNPs (attributed to recombination by the authors), as compared to free-living organism that often undergo variable selection conditions due to environmental changes, including seasonal changes (e.g., inside a host is a more stable environment, by comparison, likely).

***Again, please see our response above and our new analysis on the impact of selection on homoplasies.***

Line 213-214. Please see the fragmented speciation model in PNAS. The authors are apparently missing some key literature on the topic.

***We did omit to cite this relevant study. We have now added this citation in the revised manuscript (please see lines 285-288).***

Line 292. How this method differs from more traditional ANI e.g., using the FastANI tool (Jain et al, Nat. Comm. 2018)? Will be nice to include so readers can make direct comparisons.
***The methods are very similar in concept. CNI metrics are slightly more stringent since core genes are usually evolving slightly slower than accessory genes. Additional information on this point has been added in the revised manuscript in lines 354-359.***

**Reviewer #2:**

Diop et al present a comprehensive analysis of recombination rates in bacteria. They use a suite of methods to infer the frequency of recombination in different bacterial species, and they ultimately show that the recombination can occur across a wide range of nucleotide identities (90-98%). Remarkably, they also find evidence of "introgression" across much greater genetic distances, indicating that recombination-driven gene flow may occur even across fairly divergent lineages.  
It's difficult to overstate the importance of this work given the critical role of recombination in bacterial evolution coupled with the difficulty microbiologists have had in defining species based on gene flow. The authors also put forward a convincing argument for how to define "natural" species boundaries in bacteria using core concepts from the biological species concept (BSC) that both microbiologists and evolutionary biologists will find appealing. Most species definitions have been based on quasi-arbitrary cutoffs that are naturally quite simplistic (Jain et al, Nature Communications, 2018). Most everyone recognized that these one-size-fits-all cutoffs are not appropriate in all situations but given the daunting challenge of evaluating recombination rates across the TOL it has seemed reasonable to use this as an estimate. Diop et al present detailed analyses that reshape these perspectives and provide much-needed quantitative information on recombination rates, natural species boundaries, and even the fascinating but underappreciated role of introgression. This work will be an important foundation for future studies and will be of great interest to microbiologists and evolutionary biologists alike. I predict it will be highly cited.
***We thank the Reviewer for their comments on our work.***

I have no major concerns or revisions, but I do have some suggestions for clarifications or possible additional text inclusion below:

-This work has important implications for pan-genomics. Pangenomics has become a subject of intense debate recently (see <https://doi.org/10.1038/nmicrobiol.2017.40> and associated discussion) but it isn't even clear how to define pangenomes using current methods. Usually, researchers rely on the 95% identity threshold or other metrics not based on gene flow, but as this work shows, these cutoffs can lead to species-clusters that include distinct populations with no gene flow (and inflate pangenome sizes) or fail to capture the full diversity of a pangenome when recombination occurs across a greater genetic distance. This is just a suggestion, but if the authors do not feel that it detracts from the overall message of their study too much it may be worth including a few sentences outlining the implications of their findings for defining pangenomes more rigorously. In the future it will be important to use the BSC-defined groups here to explore patterns of pangenomic variability and see if this changes any previous conclusions in this area.

***We couldn't agree more. The accurate definition of bacterial species has implications for pan-genome analyses and other studies. We have now added a brief note on this point in the revised manuscript (lines 293-294).***

-The framework used here for identifying h/m ratios is quite impressive, especially regarding how the simulations were done to identify expected patterns for clonality for each species. This is a nontrivial task. I understand this workflow may not easily fit into a user-friendly pipeline, but in the future, I encourage the authors to find a way to make their methods a bit more accessible so that others can easily define BSC-clusters from their own input genomes. Some of these methods are already available (e.g. CoreCruncher) so it's possible the authors are already doing this, but I just wanted to stress the importance of accessible approaches to calculating h/m ratios and introgression given their broad implications for species definitions and pangenomics. Many researchers simply won't be able to perform the multiple steps, especially the simulations, on their own.
***We thank the Reviewer for their appreciation of our efforts (and for the efforts of our computer). We are currently working on making these tools accessible and integrated into a user-friendly pipeline. Unfortunately, this represents a lot of work in itself and we are hoping to have this tool available relatively soon.***

-is it possible some homoplasic alleles derive from low-complexity regions, rather than true recombination events? Or perhaps the simulations would account for this?
***We apologize but we are not sure to understand what the Reviewer means by this comment. Our simulations are accounting for the nucleotide composition of the different genomes, but also for other sources of heterogeneity (e.g. different substitution rate across codon positions, transition/transversion ratios). Low-complexity regions could be an issue by generating poor-quality alignments. However, all of our analyses were conducted on coding sequences (the core genome), and we do not expect to encounter low-complexity regions in these genes.***

-Line 111- awkward wording- suggest change to "not all endosymbiont species were found…"

***Corrected. “all endosymbiont species were not found…” was changed to "not all endosymbiont species were found…" in line 115 in the revised manuscript as suggested.***

-Line 118-120- I'm not sure I agree with the logic that endosymbionts are essential to their host and therefore escape extinction, because many endosymbionts do go extinct and are replaced (<https://doi.org/10.1038/s41396-017-0024-6>). Perhaps clonality coupled with population bottlenecks is actually a recipe for extinction, and these endosymbionts just haven't gotten there yet?
***The Reviewer is correct; our wording was imprecise. We did not mean to suggest that endosymbionts cannot go extinct, but rather, that they can substantially extend their survival in time thanks to the benefits they confer to their hosts. We have clarified the text (please see lines 141, and 143-145).***

-Line 133- where does 2446 come from? I understood after checking the methods, but perhaps a short description of where these genomes come from would help improve the logical flow here.
***A description has been added in lines 157-159 in the revised manuscript.***

-Line 156- add cites for the E. coli/Shigella issue
***The citations have been added in the revised manuscript line 187, citations 33-35.***

-Line 181- "more similar" - what about cases where all comparisons are 100 percent identical? Would that not also represent very-recent introgression?
***We agree with the Reviewer, but we decided to set a more stringent criterion to define introgression since this could theoretically be problematic for the pairs of candidate/reference species that are closely related. For such cases, it is possible—although unlikely—that these identical DNA segments are the result of vertical evolution rather than introgression. In general, we tried to avoid any bias that could lead us to over-estimate the amount of introgression in our analysis.***

-Line 187-189- this is a very important statistic, but it could be worded more clearly. Can it be re-worded so that authors get a more natural impression of the scale of introgression? Perhaps the percent of species that had introgression levels exceeding 1 percent of their genomes? And Figure S6 seems to be quite important for making the point that introgression is pervasive- perhaps this should be in the main text?
***We have clarified the text to make these statistics more explicit lines 218-219. We have also integrated Figure S6 in the main text (now Figure 3 in the revised manuscript).***

-Something about the taxonomic scale of introgression may be useful for readers (i.e., cross-genus or cross-family?), even if taxonomy is a mess and encompasses different levels of diversity across groups.
***We are now discussing the taxonomic distribution of species with signs of introgression and have added a new supplementary figure (please see lines 2239-226 and Figure S7 in the revised manuscript).***

-The discussion of MEPS is quite fascinating and adds an important mechanistic element to this study. This is key because it elevates the work compared to other purely data-driven studies on the subject that have promoted the 95% ID threshold. It shows the authors have thought quite deeply about this subject from a microbial standpoint.
***We thank the Reviewer for this comment.***

-Figure 3C is quite striking. For par C, how can a genome have an introgression score near 50% and a percent identity >95% ID but still be considered part of a different BSC-species? It seems like this is a huge amount of gene flow between quite similar genomes, so it would constitute enough recombination to permit classification within the same species. Is this because the h/m was still not higher than one would expect due to simulations? I suppose closely related but distinct species may still have high nucleotide identity, but without comparing the h/m and Si it isn't possible to tell whether this similarity is because of introgression between species or simply similarities that persist due to recent shared ancestry between separate species?
***The Reviewer is correct; in general, these cases of highly related species with high levels of introgression are indeed cases where there is a sharp drop in h/m ratio, but the h/m ratio remains relatively high. This indicates that, although gene flow is still strong between the two species, it is much less so than observed within the reference species. We found three cases where the species shared >95% genome identity and presented high levels of introgression:***

***i) Vibrio anguillarum and V. qinghaiensis share >95% genome identity and 40% of introgressed DNA. The h/m ratio went from 0.83 to 0.56.***

***ii) Neisseria meningitidis and N. polysaccharea share >95% genome identity and 39% of introgressed DNA. The h/m ratio went from 2.52 to 1.80.***

***iii) Lactobacillus helveticus and L. gallinarum share >95% identity and 52% of introgression. The h/m ratio went from 0.35 to 0.33.***

***This last case is more ambiguous since it represents a modest drop in homoplasies, but this was found highly significant in our statistical analysis. The levels of homoplasies expected from convergent mutations also account for a large fraction of these homoplasies (h/m_0_=0.24). Therefore, the relative drop­—once corrected for convergent mutations—is much more pronounced.***

***It should be noted that we did expect to find ambiguous cases and species with lower recombination rates are overall more difficult to classify with our method, particularly if they represent cases of recent or ongoing speciation. These cases are the most difficult to analyze but are exceptionally interesting since they might reveal patterns of speciation in the making. We plan to analyze these putative examples of recent or ongoing speciation in more detail in a follow-up study.***

-I enjoyed the discussion on individual species that had notable levels of gene flow and introgression. Including a few more examples throughout the text would be welcome- I found myself wondering if there was any clear link between species that experienced introgression, or species that recombined across the largest genetic distances. Of course, all this information can be looked up in the supplement, but a bit more detail in the text might be nice. This is a concise manuscript so I think there should be plenty of room.  
***A new interesting case (Neisseria) is now discussed in the revised manuscript (please see lines 231-242).***

-Line 259 - "will be compared" - seems awkward to use future tense here.
***“will be compared” has been changed to “have been compared” in the revised manuscript line 324.***

-Line 265 "your list" - awkward wording
***This was a typo. We meant "our" list. This has been corrected in the revised manuscript line 330****.*

-The methods are very detailed.
***Thank you.***

-line 282- could there be any systematic bias introduced between muscle-aligned and mafft-aligned species? I only ask because I have noticed differences in the alignments provided by these tools,
though this was mainly an issue for divergent sequences.
***Like the Reviewer wrote, muscle and mafft are only expected to provide different alignments for noisy datasets containing highly divergent sequences. Protein alignments at this level of relatedness are not expected to yield differences between the two tools. We had to use two different tools due to the limitation of muscle when dealing with very high numbers of sequences, and we did not anticipate this issue when we initiated this study.***

**Reviewer #3:**

The authors present a very interesting and well-conceived analysis in order to better quantify the extent to which gene flow shapes bacterial genomes. Importantly, this analysis is not limited to a small subset of taxa, but rather spans most of the known bacterial taxonomy and thus represents a significant advance in the fields of microbial evolution and bacterial speciation. I think it would be of great interest to biologists in a more general sense, as it provides further evidence that evolutionary theory built for sexually-reproducing organisms can be broadly applied to bacteria.

***We thank the Reviewer for his/her appreciation of our work.***

That said, I have a few concerns and questions regarding the authors' methodology.

Major points:
1) From my reading, I'm not sure that the lead statistic that 10% of analyzed species are truly clonal is fully justified. The authors use a leave-one-out approach to account for uncertainty in the observed h/m ratio, but this does not account for uncertainty in the "expected" h/m ratio generated from simulations. As I understand it, only one simulation for each species (the simulation with the closest CNI to the full dataset) was chosen to get a point estimate of the expected h/m ratio. How much variance is there in the h/m ratio for a less restricted subset of the simulations and can this be used to derive some empirical confidence intervals for the fraction of analyzed species that are truly clonal?
***Simulating these datasets and their subsequent h/m statistics was computationally expensive and generating a range of h/m values would require many additional simulations. We do not think that repeating these simulations would necessarily reflect biological variations. Instead, we originally opted to use a completely different approach by analyzing patterns of Linkage Disequilibrium in addition to the simulations. We believe that using two different approaches represent a more thorough analysis than generating additional simulations. We did not conduct the additional simulations suggested by the Reviewer since it would substantially prolong the time required to revise the manuscript (several months); however if the Reviewer and the Editor believe that these extra simulations need to be conducted, this can be done (with a time extension).***

2) Related to point 1, how do the authors explain cases where the observed h/m is significantly lower than the simulated h/m? (E.g., supplementary table 2, Xanthomonas_euvesicatoria)? Is it possible to be "more clonal" than expected or is there something that the simulations are not fully capturing about clonal evolution?
***Our simulations have been conducted while mimicking the real datasets as closely as possible. To this end we had to estimate multiple parameters from the real datasets, and it is possible that some of these estimated parameters are slightly off. While this is not expected to have a substantial impact for most species, we observed that several species with very little genomic diversity (low levels of polymorphism) tend to be more difficult to estimate accurately. The species mentioned by the Reviewer (Xanthomonas euvesicatoria) is a good example of this. The genomes of this species present 99.9% nucleotide identity on average. However, we verified that this did not impact our prediction of clonal species: as shown in Figure S2C, we did not observe that the inferred clonal species tend to be less diverse than the non-clonal ones. Nevertheless, we have now added a comment in the text lines 108-111 to discuss the lower accuracy of our simulations for those species with low levels of polymorphisms.***

3) Much of the analysis here depends on the definition of the core genome, which in turn depends on species definitions to begin with. To what extent are h/m estimates from the core genome of a species that has a CNI of ~98% comparable to the h/m estimates for species with a CNI of say, 90%? I don't have a particularly compelling suggestion here, but I think this point deserves more discussion.
***Core genome sizes vary depending on the size of the dataset and the divergence of the genomes. Our experience and previous works suggest that h/m estimates do not vary substantially when analyzing different numbers of core genes (at least dozens). It is for this reason that we did not build a single core genome for each genus since it would result in a much smaller core genome. Ultimately, the ability to reconstruct core genomes is always a limiting factor for most analyses in microbial genomics, but previous works have shown that gene flow does not vary tremendously along the core genome and that even analyzing a dozen of genes provides a robust estimate of gene flow (Didelot et al., BMC Genomics 2012; Yahara et al., MBE 2016). Our core genomes are typically composed of hundreds or thousands of core genes (See Table S3). We are now discussing this point lines 163-167.***

4) Why was only one genome from a candidate species used when defining the core genome of each candidate/reference species pair? In the case where CNI within a candidate species was relatively low, wouldn't one expect that the specific representative chosen would have a large effect on the inference of the core genome for the candidate/reference pair?
***It is true that the set of chosen genomes will always have some impact when building the core genome. Since we were facing some computational limitations in this study, we decided to introduce a single genome of candidate species to lower the computational burden of the analysis. Moreover, our previous studies have also shown that testing a single genome at a time relative to a reference species provides more robust statistical signal (see Bobay & Ochman, GBE 2017). Again, we are not concerned by the variation in the size and composition of the core genomes since h/m estimates tend to be very robust when averaged across hundreds or thousands of genes (see previous comments).***

5) In Figure 3C, is there an explanation for why the observed recent introgression score is actually lower than expected at high identity?
***This point was also raised by another Reviewer, and the confusion is due to a lack of clarity in the text. In brief, the number of identical fragments to originate from vertical evolution is not trivial to estimate, since it depends on the divergence of species and the levels of polymorphisms within each species. We only expect to find such identical tracts when species are closely related and when a reference species presents very low levels of polymorphisms. The expected line represents the upper estimate of identical 100bp expected to be inherited vertically (estimated from a pair of simulated genomes). We have now clarified what the expected line actually represents (see lines 249-259 and Figure 4c).***

Minor points:
1) There are a few instances where it seems a term is defined after it is first used or should be used. For example, in line 131, does "every species" mean "every candidate species"? And in lines 140-144, h/m0 is introduced a little before it is described.

***Thank you for catching this. We have now fixed these issues throughout the text. h/m_0_ has been described lines 172-173 in the revised manuscript as suggested.***

2) It was initially unclear to me that the introgression analysis was only conducted on candidate species that did not get assigned to a BSC reference species.

***This point has been clarified in the revised manuscript (please see lines 208-209).***

3) Figure 3C: Is the y-coordinate of the red line meant to be the MEPS frequency and not the introgression score?

***The Reviewer is right, the y-coordinate of the red line is meant to be the* *MEPS frequency. We have added a y-axis for the red line (Please see figure 4C in the revised manuscript)*.**

**Second round of review**

**Reviewer 1**

The overall conclusions of the abstract and the spin of the paper, in general, clearly imply that gene flow is envisioned by the authors as the mechanism for species cohesion (or the 95% ANI clusters). But the authors do not prove that recombination is random enough across the genome to serve as a force of cohesion; in fact, in their response, they admit that they have not tested/quantified this. So, the paper just shows/assesses gene flow, not a direct assessment of the biological/sexual species concept. This has to come more clear, up front (e.g., in the abstract), in my opinion. I would be ok if the authors just say/conclude that some species exchange (core) genes and a few others not; but for suggesting that this is the mechanism of species cohesion (as they do), I don’t believe the authors have offered adequate, quantitative results in support of this claim, in this or their previous papers. Also, core genes could be under positive (adaptive) selection such as flagella genes and ARG (when core)...the core genes that are more likely to NOT be under positive selection are ribosomal proteins, DNA/RNA polymerase, etc. Do the authors see a difference between such functional categories of core genes in terms of frequency of gene flow? In general, it seems like a missed opportunity that the authors do not examine (and report) on specific gene functions under recombination. Further, positive selection could drive the HGT (and subsequent recombination) of an already adapted allele that has therefore few non-synonymous vs. synonymous substitutions; that is, the recombined gene does not have to have more non-synonymous substitutions relative to the native allele. In fact, most bacterial genes should be expected to be already well adapted/fit for their function since genes with Dn/Ds >1 are rare in any genomes, making up typically <1% of total genes. So, why the horizontal transfer and recombination of such genes should have been expected to give a higher frequency of non-synonymous to synonymous substitutions? Not clear to me as written. Accordingly, I remain behind my original comment that the authors’ model does not include selection and thus, it is probably unrealistic. I believe the author ought to discuss this issue (e.g., selection and its consequences) in more detail and better qualify their statements and conclusions. Finally, have the authors applied their approach to higher eukaryotic genomes, which are known to be sexual, and seen similar results? Without this data, this conclusion in the abstract is not fully justified “…that bacterial evolution and speciation are likely shaped by similar forces driving the evolution of sexual organisms”. (This is also a clear example of the authors clearly implying species cohesion by recombination; related to my major point above).


From authors’ responses:

The idea behind selection driving convergent mutations (and the studies cited) is based
on the assumption of adaptive evolution/selection, not purifying selection. It should be noted
that the studies cited by the Reviewer are cases of bacteria put under very strong selective
pressures (e.g. antibiotic resistance) and in very simplified environments and the cell lines of
Lenski are clearly maladapted to their environment.

>I don’t think the bacteria the authors study are necessarily different from the Campylobacter case I mentioned, e.g., many of the species analyzed are pathogens or opportunistic pathogens, under antibiotic selection just like Campylobacter. Let me also point out that Campylobacter jejuni and C. coli are among the most recombinogenic bacteria found to date.

In contrast, the homoplasies in our study have been detected in the core genome, where genes are evolving under purifying selection. Adaptive evolution is not expected to be strong or common in these genes.

>It depends on the type of core genes; for ribosomal proteins and DNA/RNA polymerases, yes, probably this is true but for flagella genes, ARG (if/when core), metabolic functions or regulation and environmental sensing, I don’t think what the authors claim is necessarily true. Also, it would be important to mention how many homoplacies were found; that is, to offer a more quantitative view e.g. are we talking about a couple homoplacies examine or hundreds? I don’t think I saw this information anywhere (and I could not find Supp. Fig. S3).

It is also very unlikely that adaptation would occur through similar independent mutations in natural conditions where selective pressures are complex and multiple.
>Are there any data to support this claim?

Bacterial strains have different genotypes and gene content, which implies that the same mutation is unlikely to provide the same adaptive advantage in different genomic backgrounds.
>MAGs that represent the average population genomes are often very stable in gene content, even between samples or time points. I believe what the authors claim is for the non-core, auxiliary gene content (which is apparently not their focus since they target core genes).

Nevertheless, the point raised by the Reviewer can be tested with our dataset. It is
expected that adaptive convergence would affect non-synonymous sites rather than
synonymous ones. Although synonymous sites can experience some selection, those are much
weaker and are not the primary target of adaptive evolution. In addition, selective pressures
acting on synonymous sites are typically associated with codon usage and/or nucleotide
composition of an entire gene overall. This last point indicates that selection at synonymous
positions is not expected to affect a particular site, but, rather, the overall sequence of the
gene.
>As I report above, the great majority of genes in the genome are already well adapted/fit and, as a result, they typically show Dn/Ds ~0.2-0.3…So, why the horizontal transfer and recombination of such genes is expected to give a higher frequency of non-synonymous to synonymous substitutions?

**Reviewer 2**

The authors have done a nice job revising the manuscript. It was already in pretty good shape, and now it has been improved even further. All of my concerns have been addressed. This is an important study that will inform the field for many years to come.

I do not agree with the concern raised by Reviewer 1 regarding the role of selection for explaining the distribution of homoplasic alleles. This is a complex topic, and to some extent is is a rehash of classic paradoxes like Haldane’s dilemma, which is clearly outside the scope of this study. But most evolutionary genomicists would likely agree that neutral or nearly neutral mutations are responsible for the large level of allelic polymorphism in populations and between species. Indeed, the additional analyses by Diop and colleagues support this consensus view. This is not to say that selection is not important, but rather to say that invoking selection as a mechanism to explain thousands of homoplasic alleles is a bit beyond the pale. And given the currently-accepted methods are to simply ignore all of this and use 95% identity as the threshold, it is hard to see how the method of Diop et al could not be viewed as a vast improvement.

**Reviewer 3**

I am satisfied with the responses that the authors have given to my comments and look forward to wider discussion of this manuscript in the community. After reading the authors' response, I do not believe that the additional simulations I suggested in my comment are necessary. The revised work sufficiently supports its conclusions and includes some important clarifications and caveats.

**Authors’ response to reviewers**

Reviewer reports:

Reviewer #1:  The overall conclusions of the abstract and the spin of the paper, in general, clearly imply that gene flow is envisioned by the authors as the mechanism for species cohesion (or the 95% ANI clusters). But the authors do not prove that recombination is random enough across the genome to serve as a force of cohesion; in fact, in their response, they admit that they have not tested/quantified this. So, the paper just shows/assesses gene flow, not a direct assessment of the biological/sexual species concept. This has to come more clear, up front (e.g., in the abstract), in my opinion. I would be ok if the authors just say/conclude that some species exchange (core) genes and a few others not; but for suggesting that this is the mechanism of species cohesion (as they do), I don't believe the authors have offered adequate, quantitative results in support of this claim, in this or their previous papers.
***We thank the Reviewer for his/her comments. Our results support a role of gene flow in speciation based on the patterns of gene flow relative to genomic divergence. Our point is that gene flow is a mechanism that has been clearly identified as a force of genetic cohesion in sexual organisms and we are simply suggesting that the same mechanisms could be at play in bacteria. We are now providing evidence that gene flow is random since it is impacting virtually every single gene in the core genome. Nevertheless, we have modified our text according to the comments of the Reviewer.***

Also, core genes could be under positive (adaptive) selection
such as flagella genes and ARG (when core)...the core genes that are more likely to NOT be under  positive selection are ribosomal proteins, DNA/RNA polymerase, etc. Do the authors see a difference between such functional categories of core genes in terms of frequency of gene flow? In general, it seems like a missed opportunity that the authors do not examine (and report) on specific gene functions under recombination. Further, positive selection could drive the HGT (and subsequent recombination) of an already adapted allele that has therefore few non-synonymous vs. synonymous substitutions; that is, the recombined gene does not have to have more non-synonymous substitutions relative to the native allele. In fact, most bacterial genes should be expected to be already well adapted/fit for their function since genes with Dn/Ds >1 are rare in any genomes, making up typically <1% of total genes. So, why the horizontal transfer and recombination of such genes should have been
expected to give a higher frequency of non-synonymous to synonymous substitutions? Not clear to me as written. Accordingly, I remain behind my original comment that the authors' model does not include selection and thus, it is probably unrealistic. I believe the author ought to discuss this issue (e.g., selection and its consequences) in more detail and better qualify their statements and conclusions.

***The point raised by the Reviewer regarding positive selection is confusing to us. As stated by the Reviewer, very few genes are under positive selection in the core genome and therefore this mechanism could not explain the patterns of homoplasies that we observed (thousands of homoplasic alleles across hundreds or thousands of genes). The Reviewer is right about the fact that, in theory, gene regions under positive selection do not necessarily yield an excess of non-synonymous substitutions. However, this type of genomic signatures has been analyzed in many studies and to the best of our knowledge, pervasive positive selection has never been reported (also see our point below). The text has been revised as suggested by the Reviewer.***

Finally, have the authors applied their approach to higher eukaryotic genomes, which are known to be sexual, and seen similar results? Without this data, this conclusion in the abstract is not fully justified "…that bacterial evolution and speciation are likely shaped by similar forces driving the evolution of sexual organisms". (This is also a clear example of the authors clearly implying species cohesion by recombination; related to my major point above).

***We did previously show that our approach can identify species borders in sexual organisms (Bobay and Ochman GBE 2017). This has been added to the text.***

From authors' responses:

The idea behind selection driving convergent mutations (and the studies cited) is based
on the assumption of adaptive evolution/selection, not purifying selection. It should be noted
that the studies cited by the Reviewer are cases of bacteria put under very strong selective
pressures (e.g. antibiotic resistance) and in very simplified environments and the cell lines of
Lenski are clearly maladapted to their environment.

>I don't think the bacteria the authors study are necessarily different from the Campylobacter case I mentioned, e.g., many of the species analyzed are pathogens or opportunistic pathogens, under antibiotic selection just like Campylobacter. Let me also point out that Campylobacter jejuni and C. coli are among the most recombinogenic bacteria found to date.
***Very few genes in bacterial genomes are involved with antibiotic resistance and virulence and the vast majority of these genes are not part of the core genome (for instance, see Rocha 2008 Annual review of genetics).***

In contrast, the homoplasies in our study have been detected in the core genome, where genes are evolving under purifying selection. Adaptive evolution is not expected to be strong or common in these genes.

>It depends on the type of core genes; for ribosomal proteins and DNA/RNA polymerases, yes, probably this is true but for flagella genes, ARG (if/when core), metabolic functions or regulation and environmental sensing, I don't think what the authors claim is necessarily true. Also, it would be important to mention how many homoplacies were found; that is, to offer a more quantitative view e.g. are we talking about a couple homoplacies examine or hundreds? I don't think I saw this information anywhere (and I could not find Supp. Fig. S3).

***The Reviewer is right; we did not provide this data, and this could explain the comments of the Reviewer regarding the potential role of positive selection. We identified thousands of homoplasies (average of 65,670) for each bacterial species and homoplasies were virtually found in every single core gene (including genes encoding for ribosomal proteins). This corresponds to an average of 35 homoplasies per core gene across species. We would like to underline that it is therefore extremely improbable that all or most of these homoplasies are the result of positive selection or nearly every single gene would be evolving under positive selection. We have now added these metrics for each reference species in Additional file 3 (Table S2).*** ***This point has also been added in the text.***

It is also very unlikely that adaptation would occur through similar independent mutations in natural conditions where selective pressures are complex and multiple.
>Are there any data to support this claim?

***Many studies have shown that even the same ancestral strain evolves predominantly through independent mutations despite being exposed to the same experimental conditions. See for instance Whichman at al. Science 1999, Cooper et al. PNAS 2003 and Fong et al. Genome Res 2005. These independent evolutionary paths are expected to be even more different when occurring within different strains and under diverse environmental conditions.***

Bacterial strains have different genotypes and gene content, which implies that the same mutation is unlikely to provide the same adaptive advantage in different genomic backgrounds.
>MAGs that represent the average population genomes are often very stable in gene content, even between samples or time points. I believe what the authors claim is for the non-core, auxiliary gene content (which is apparently not their focus since they target core genes).

***The Reviewer is correct, we were mentioning the high variability of accessory genes across bacterial strains. Although our study is indeed focusing on the core genome, our point here was to underline that each strain typically possesses a unique gene repertoire, and that the presence of some accessory genes is known to contribute to phenotypic differences. It is therefore expected that through the diversity of phenotypes and genomic backgrounds across strains that a given mutation in a given core gene may not have the same phenotypic impact across strains.***

Nevertheless, the point raised by the Reviewer can be tested with our dataset. It is
expected that adaptive convergence would affect non-synonymous sites rather than
synonymous ones. Although synonymous sites can experience some selection, those are much
weaker and are not the primary target of adaptive evolution. In addition, selective pressures
acting on synonymous sites are typically associated with codon usage and/or nucleotide
composition of an entire gene overall. This last point indicates that selection at synonymous
positions is not expected to affect a particular site, but, rather, the overall sequence of the
gene.
>As I report above, the great majority of genes in the genome are already well adapted/fit and, as a result, they typically show Dn/Ds ~0.2-0.3…So, why the horizontal transfer and recombination of such genes is expected to give a higher frequency of non-synonymous to synonymous substitutions?
***We were looking for signatures of positive selection and dN/dS is one approach to do so. We agree that it does not necessarily prove the lack of positive selection. Nevertheless, the fact that we report thousands of homoplasies across core genes would require pervasive and nearly constant positive selection in bacteria. To the best of our knowledge, population genetics studies focusing on the standing variation of bacterial populations are not consistent with a scenario of massive genome-wide positive selection (see Touchon et al. PLoS Genet 2009 for instance). This point is discussed in the manuscript.***

Reviewer #2: The authors have done a nice job revising the manuscript. It was already in pretty good shape, and now it has been improved even further. All of my concerns have been addressed. This is an important study that will inform the field for many years to come.

I do not agree with the concern raised by Reviewer 1 regarding the role of selection for explaining the distribution of homoplasic alleles. This is a complex topic, and to some extent is is a rehash of classic paradoxes like Haldane's dilemma, which is clearly outside the scope of this study. But most evolutionary genomicists would likely agree that neutral or nearly neutral mutations are responsible for the large level of allelic polymorphism in populations and between species. Indeed, the additional analyses by Diop and colleagues support this consensus view. This is not to say that selection is not important, but rather to say that invoking selection as a mechanism to explain thousands of homoplasic alleles is a bit beyond the pale. And given the currently-accepted methods are to simply ignore all of this and use 95% identity as the threshold, it is hard to see how the method of Diop et al could not be viewed as a vast improvement.

***We thank the Reviewer for his/her review and for commenting on the concerns of Reviewer #1.***

Reviewer #3: I am satisfied with the responses that the authors have given to my comments and look forward to wider discussion of this manuscript in the community. After reading the authors' response, I do not believe that the additional simulations I suggested in my comment are necessary. The revised work sufficiently supports its conclusions and includes some important clarifications and caveats.

***We thank the Reviewer for his/her comments and for taking the time to evaluate our work.***
